# Synthesis and Preclinical Evaluation of 22-[^18^F]Fluorodocosahexaenoic Acid as a Positron Emission Tomography Probe for Monitoring Brain Docosahexaenoic Acid Uptake Kinetics

**DOI:** 10.1021/acschemneuro.3c00681

**Published:** 2023-12-04

**Authors:** Marlon Vincent V. Duro, Juno Van Valkenburgh, Diana E. Ingles, Jenny Tran, Zhiheng Cai, Brandon Ebright, Shaowei Wang, Bilal E. Kerman, Jasmin Galvan, Sung Hee Hwang, Naomi S. Sta Maria, Francesca Zanderigo, Etienne Croteau, Stephen C. Cunnane, Stanley I. Rapoport, Stan G. Louie, Russell E. Jacobs, Hussein N. Yassine, Kai Chen

**Affiliations:** † Department of Radiology, Keck School of Medicine, University of Southern California, Los Angeles, California 90033, United States; ‡ Department of Medicine, Keck School of Medicine, University of Southern California, Los Angeles, California 90033, United States; § Alfred E. Mann School of Pharmacy and Pharmaceutical Sciences, University of Southern California, Los Angeles, California 90089, United States; ∥ Department of Entomology and Nematology and UC Davis Comprehensive Cancer Center, University of California, Davis, California 95616, United States; ⊥ Zilkha Neurogenetic Institute, Keck School of Medicine, University of Southern California, Los Angeles, California 90033, United States; # Department of Psychiatry, Columbia University, New York, New York 10032, United States; ∇ Molecular Imaging and Neuropathology Area, New York State Psychiatric Institute, New York, New York 10032, United States; ○ Sherbrooke Center for Molecular Imaging, University of Sherbrooke, Sherbrooke, QC J1H 4C4, Canada; ◆ Research Center on Aging, Department of Medicine, University of Sherbrooke, Sherbrooke, QC J1H 4C4, Canada; ¶ National Institute on Alcohol Abuse and Alcoholism, Bethesda, Maryland 20892-9304, United States

**Keywords:** positron emission tomography, radiofluorination, docosahexaenoic acid, incorporation coefficient, polyunsaturated fatty acid

## Abstract

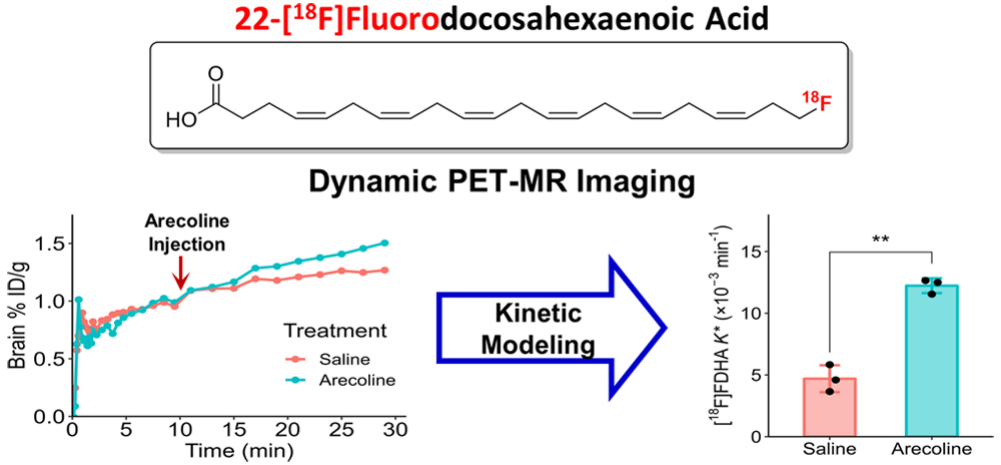

Docosahexaenoic acid [22:6(*n*-3), DHA], a polyunsaturated fatty acid, has an important role in regulating neuronal functions and in normal brain development. Dysregulated brain DHA uptake and metabolism are found in individuals carrying the APOE4 allele, which increases the genetic risk for Alzheimer’s disease (AD), and are implicated in the progression of several neurodegenerative disorders. However, there are limited tools to assess brain DHA kinetics *in vivo* that can be translated to humans. Here, we report the synthesis of an ω-radiofluorinated PET probe of DHA, 22-[^18^F]fluorodocosahexaenoic acid (22-[^18^F]FDHA), for imaging the uptake of DHA into the brain. Using the nonradiolabeled 22-FDHA, we confirmed that fluorination of DHA at the ω-position does not significantly alter the anti-inflammatory effect of DHA in microglial cells. Through dynamic PET-MR studies using mice, we observed the accumulation of 22-[^18^F]FDHA in the brain over time and estimated DHA’s incorporation coefficient (K*) using an image-derived input function. Finally, DHA brain K* was validated using intravenous administration of 15 mg/kg arecoline, a natural product known to increase the DHA K* in rodents. 22-[^18^F]FDHA is a promising PET probe that can reveal altered lipid metabolism in APOE4 carriers, AD, and other neurologic disorders. This new probe, once translated into humans, would enable noninvasive and longitudinal studies of brain DHA dynamics by guiding both pharmacological and nonpharmacological interventions for neurodegenerative diseases.

## Introduction

Docosahexaenoic acid [22:6(*n*-3), DHA] is the most abundant polyunsaturated fatty acid (PUFA) in the brain^1^ where it regulates several important processes and serves as a precursor to bioactive mediators to resolve inflammation in neurons, microglia, and endothelial cells.^2,3^ However, the capacity of the brain to synthesize DHA locally is low, and the uptake of DHA from circulating lipid pools is arguably essential to maintaining homeostatic levels.^2,4−6^ Brain DHA uptake and metabolism are affected by various factors that are implicated in the progression of neurodegenerative disorders such as Alzheimer’s disease (AD).^7,8^ One factor that influences the metabolism and the production of bioactive lipid mediators in the brain is the apolipoprotein E4 (APOE4) allele, a major genetic risk factor for AD;^9^ APOE4 affects the catabolism, transport, and esterification of DHA in the brain, leading to changes in arachidonic acid (AA) and DHA signaling cascades across the lifespan.^10−14^ These changes are associated with increased brain inflammation and amyloid deposition with APOE4 disrupting the associations of serum or plasma with brain DHA levels.^15−17^

In AD dementia, higher plasma DHA levels are associated with lower amyloid burden in individuals without APOE4 but not in those with APOE4.^15^ The relationship between plasma and cerebrospinal fluid (CSF) DHA in individuals was found to be weaker in cognitively normal APOE4 carriers compared to noncarriers.^18^ These findings suggest that APOE4 impairs the transport of DHA to the brain. The optimal dosage of DHA supplementation for AD prevention is still unclear. Some studies suggest that high doses (>2 g/day) of DHA may be needed for adequate brain bioavailability and that APOE4 carriers may have reduced response to supplementation compared to noncarriers.^18−21^ There is evidence suggesting that APOE4 status attenuates brain delivery and effects of supplemental DHA by downregulating the expression of DHA transporters^10,11^ or activating catabolic enzymes such as the calcium-dependent phospholipase A2.^22^ However, further research is needed to elucidate brain DHA transport.

Several methods have been employed to assess the uptake and turnover of DHA in the brain, the earliest example of which was the use of radiolabeled (^3^H or ^14^C) DHA infusates to investigate factors that affect the uptake and metabolism of DHA through autoradiography of post-mortem rodent brains.^10,23−27^ Other methods involve the analysis of the ratio changes of nonradioactive isotopes, such as ^2^H and/or ^13^C, in animal brain tissue using natural or synthetic DHA by mass spectrometric techniques.^28−30^ In humans, uniformly ^13^C-labeled DHA has been employed to analyze the relationship between plasma and whole-body (from analysis of CO_2_ from breath), which is affected by normal aging and the APOE4 phenotype.^14,31^

In addition to the above methods, positron emission tomography (PET) is an imaging modality that enables measurement of DHA uptake into the brain *in vivo* and has been employed to assess DHA uptake in human brains.^32,33^ Using 1-[^11^C]DHA as a PET probe, the regional and global incorporation coefficient (K*) of DHA into the human brain have been estimated, which were influenced by factors such as cerebral blood flow and unesterified DHA concentration in the plasma. We previously reported using this imaging technique that DHA K* is higher in cognitively normal APOE4 carriers during the midthirties compared to noncarriers.^34^ However, the use of 1-[^11^C]DHA as a PET probe comes with limitations, primarily its short half-life (20 min), which hinders its application in translational studies of DHA uptake. In contrast, the ^18^F radioisotope of fluorine, with its significantly longer half-life (109.8 min) compared to ^11^C, not only enables the synthesis of PET probes through multistep processes but also facilitates extending imaging procedures over the course of several hours. In recent years, the US Food and Drug Administration (FDA) has approved the use of small molecules modified with ^18^F in radiodiagnostics, emphasizing the increasing preference for ^18^F-based derivatives in the development of innovative and effective PET agents.^35^

**Figure 1:**
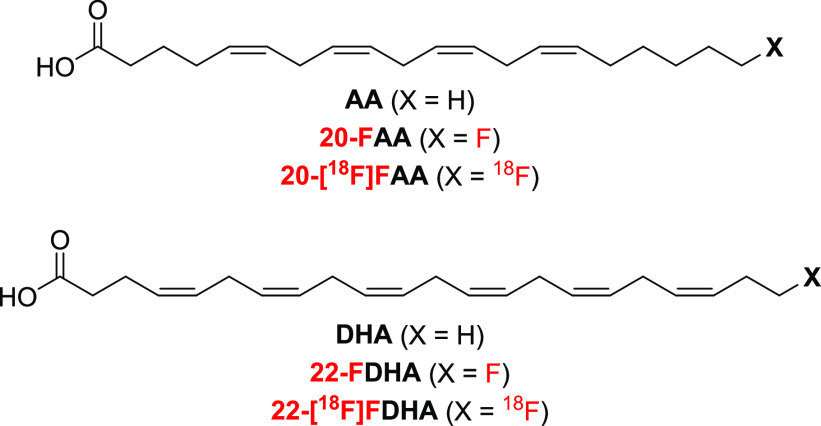
Chemical structures of polyunsaturated fatty acids AA and DHA and their ω-fluorinated analogues.

The purpose of this study was to synthesize ^18^F-labeled DHA for *in vivo* brain DHA imaging. Recently, we used 20-[^18^F]fluoroarachidonic acid (20-[^18^F]FAA, Figure 1) to evaluate AA uptake and kinetics in the brains of APOE4-targeted replacement mice through dynamic PET-MR imaging.^36^ Here, we report the synthesis of an ω-fluorinated DHA PET probe, 22-[^18^F]fluorodocosahexaenoic acid (22-[^18^F]FDHA, Figure 1). Then, we validated the observed uptake of the tracer into the brain by monitoring the changes in brain uptake kinetics through arecoline administration. The development of of the 22-[^18^F]FDHA PET probe has the potential to address the hurdles related to the short-lived 1-[^11^C]DHA PET probe. This development could yield more precise and readily applicable assessments of DHA incorporation into the brain.

## Materials and Methods

### Synthetic Procedures

#### Synthesis of 22-FDHA

**Scheme 1:**
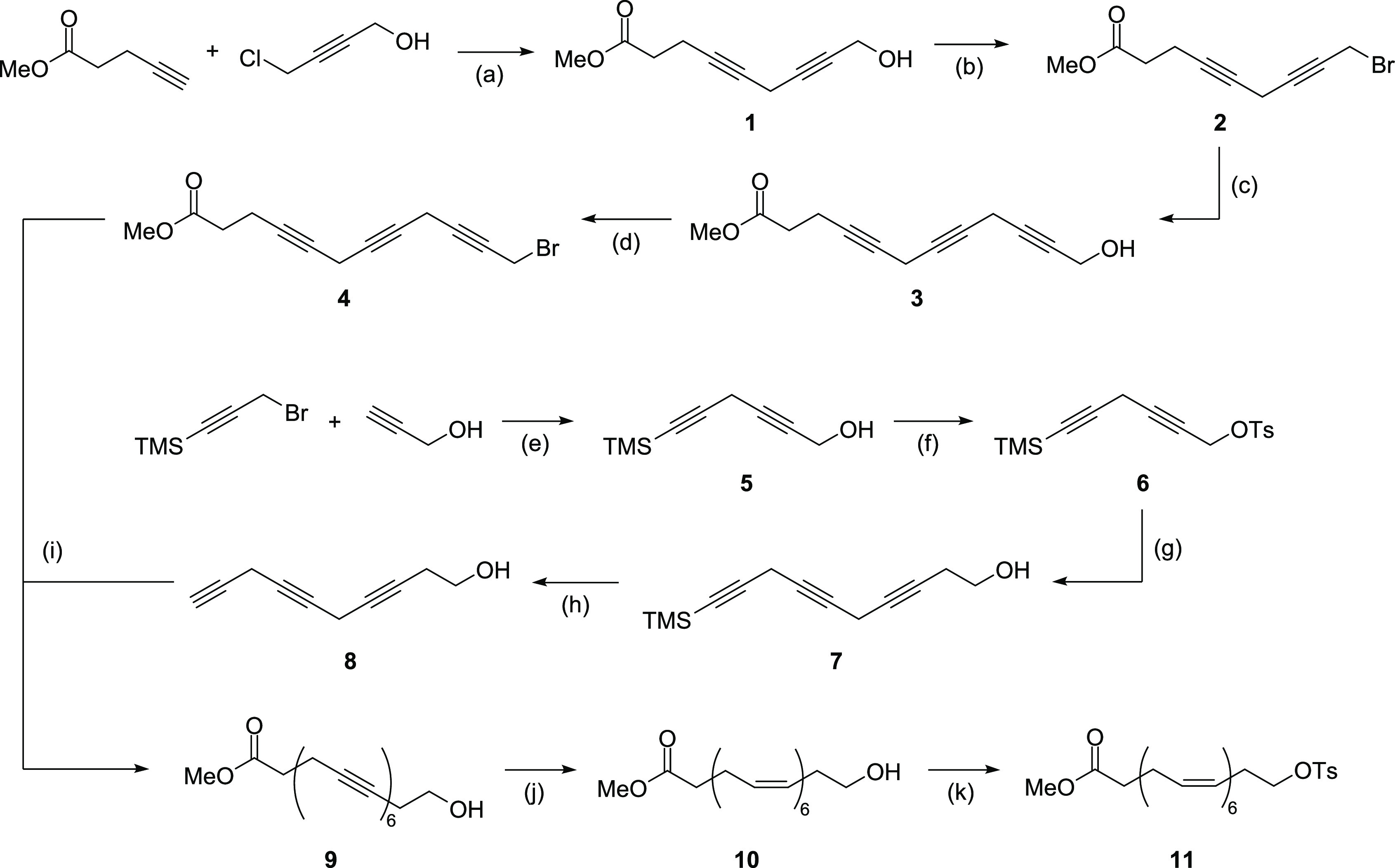
Synthesis of Fluorination Precursor **11** (a) CuI, NaI, Cs_2_CO_3_, DMF, rt, 83% yield; (b) CBr_4_, PPh_3_, DCM, 0 °C, 80% yield; (c) propargyl alcohol, CuI, NaI, Cs_2_CO_3_, DMF, rt, 65% yield; (d) CBr_4_, PPh_3_, DCM, 0 °C, 61% yield; (e) CuI, NaI, Cs_2_CO_3_, DMF, rt, 94% yield; (f) Ts_2_O, pyridine, DCM, rt, 60% yield; (g) 3-butyn-1-ol, CuI, NaI, Cs_2_CO_3_, DMF, rt, 48% yield; (h) TBAF, acetic acid, THF, 0 °C, 84% yield; (i) CuI, NaI, Cs_2_CO_3_, DMF, rt, 41% yield; (j) H_2_ (1 atm), Lindlar’s catalyst, 4:2:2:1 (v/v) 2-methyl-2-butene/EtOAc/methanol/pyridine, rt; (k) TsCl, Et_3_N, DMAP, DCM, 0 °C to rt, 13% total yield over two steps from compound **9**.

**Scheme 2:**
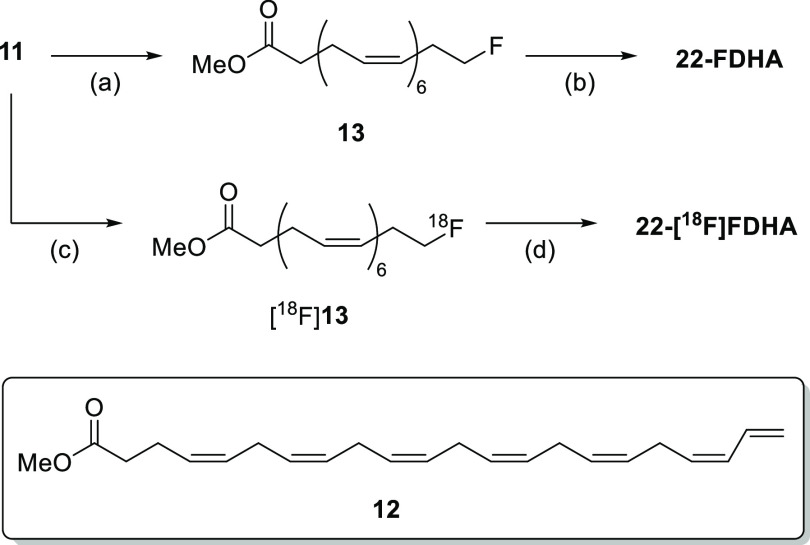
Synthesis of the Nonradiolabeled and Radiolabeled Fluorinated DHAs, 22-FDHA, and 22-[^18^F]FDHA (a) TBAF, THF, 0 °C to rt, 29% yield; (b) LiOH, THF/water, rt, 94% yield; (c) [^18^F]TBAF, MeCN, 85 °C, 20 min; (d) KOH, water/MeCN, 85 °C, 15 min.

General synthetic methods for the synthesis of a common intermediate **11** (the tosylate precursor for fluorination, Scheme 1) and synthetic methods for 22-FDHA (Scheme 2) are fully described in the Supporting Information.

#### Radiosynthesis of 22-[^18^F]FDHA

22-[^18^F]FDHA was synthesized from the labeling precursor **11** (Scheme 1) and [^18^F]fluoride ion generated by the ^18^O(p,n)^18^F nuclear reaction in a GE PETtrace 800 cyclotron, adapting a procedure from our report on the synthesis of 20-[^18^F]FAA.^36^ [^18^F]fluoride ion in [^18^O]water was transferred through a preconditioned (10 mL ethanol followed by 10 mL water) anion exchange cartridge (QMA). The retained [^18^F]fluoride was eluted into a V-vial with 0.075 M aq tetrabutylammonium bicarbonate solution (0.4 mL). Anhydrous MeCN (1 mL) was added to the V-vial, and the resulting solution of [^18^F]TBAF was azeotropically dried at 100 °C with nitrogen flow by subsequent addition of 1 mL portions of anhyd. MeCN (3×).

A solution of **11** (ca. 1.5 mg) in anhyd. MeCN (0.5 mL) was added to the residue in the V-vial, and the reaction mixture was shaken and heated at 85 °C for 20 min. After cooling for 5 min, 2 M aq KOH (0.5 mL) was added to the vial. The mixture was shaken and heated again at 85 °C for 15 min. After cooling, the mixture was then acidified with 1 M aq oxalic acid (0.75 mL) and the crude reaction mixture was purified by semipreparative reversed-phase high-performance liquid chromatography (HPLC, method C, Supporting Information). After evaporation of the fraction containing the product under reduced pressure, 22-[^18^F]FDHA was obtained. For use in the *in vivo* experiments, the 22-[^18^F]FDHA probe was formulated in 0.75 mL of freshly prepared 5 mM HEPES buffered saline containing 50 mg/mL fatty-acid-free bovine serum albumin (BSA) by sonication.

#### Partition Coefficient

The 1-octanol–phosphate-buffered saline (PBS) partition coefficient was measured at room temperature and the value was designated as log*P*. A solution of 22-[^18^F]FDHA (*ca*. 370 KBq) in 10 μL of PBS (pH = 7.4) was placed in a microcentrifuge tube containing 500 μL of PBS (pH 7.4) and 500 μL of 1-octanol. The mixture was vortexed for 3 min and then centrifuged (12,000 × *g*) for 10 min. The PBS and 1-octanol layers (150 μL of each layer) were pipetted into separate gamma counter test tubes. The radioactivity was determined by using a PerkinElmer 2480 WIZARD automatic gamma counter (PerkinElmer Inc., Waltham, MA). The partition coefficients of 1-octanol-to-PBS were calculated as log*P* = log([organic-phase cpm]/[aqueous-phase cpm]). Measurements were carried out in quintuplicate.

### Bioequivalence Assay

#### Cell Culture and Lysate Preparation

The immortalized microglial cell line BV-2 was grown and maintained in Dulbecco’s modified Eagle’s medium (DMEM, Corning, 10013CV) supplemented with 10% fetal bovine serum and 1% antibiotic-antimycotic. Seeded BV-2 cells (5 × 10^4^) were stimulated with 200 ng of lipopolysaccharide (LPS) and then treated with DHA or 22-FDHA (6 or 15 μM) for 6 h. Cells were then lysed in 60 μL of 1× RIPA buffer (Cell Signaling Technology, CST 9806) containing a protease inhibitor cocktail (Sigma, P8340), phosphatase inhibitor cocktail (Sigma, P0044), and 4× sample buffer (Bio-Rad, 1610747). The cell lysates were then boiled for 5 min and collected for Western blot.

#### Cyclooxygenase-2 (COX-2) Western Blot

Proteins from the cell lysates were separated using 4–15% Criterion TGX Precast gels (Bio-Rad, 5671085), transferred onto a nitrocellulose membrane (Bio-Rad, 1704270) and blocked with 5% fat-free milk (Bio-Rad, 1706404) for 2 h at room temperature. The membranes were then incubated with the primary antibody diluted in 5% BSA at 4 °C overnight. The membranes were then washed (3 × 5 min) with Tris-buffered saline with 0.1% Tween-20 (TBS-T) and incubated with horseradish peroxidase (HRP)-conjugated secondary antibody for 30 min at room temperature. After washing with TBS-T (3 × 5 min), the membranes were imaged using a Fujifilm LAS-4000 imager system, and protein was detected using chemiluminescent HRP substrate (Millipore, WBKLS0500). GelQuant.NET software was used for densitometric quantification. The following antibodies and dilution factors were used: COX-2 rabbit antibody (CST,12282; 1:1000), actin rabbit antibody (CST, 4970; 1:1000), and HRP-linked antirabbit IgG (CST, 7074; 1:2000).

### 22-[^18^F]FDHA *In Vitro* Stability

#### Formulation Stability

A solution of *ca*. 37 MBq of 22-[^18^F]FDHA in 0.25 mL of HEPES buffered saline (5 mM) containing fatty-acid-free BSA (50 mg/mL) was incubated at room temperature for 5 h. The sample was diluted with water and analyzed by HPLC (method A, Supporting Information) to determine the stability of the probe. Measurements were performed in duplicate.

#### Stability in Mouse Serum

Solutions of *ca*. 9.25–74 MBq of 22-[^18^F]FDHA in mouse serum (250 μL) were shaken at 37 °C for 1, 2, 4, 6, and 8 h, respectively. After addition of trifluoroacetic acid (TFA, 10 μL), each mixture was vortexed for 3 min and centrifuged for 10 min (12,000 × *g*). The resulting supernatants were analyzed by HPLC (method A, Supporting Information). Measurements were performed in duplicate.

### Animals

All animal studies were approved by the Institutional Animal Care and Use Committee at the University of Southern California. The female C57BL/6J mice (6-month-old, weighing 21–30 g) used in the study were bred in the USC animal facility. Animals were housed with standardized 12 h light and dark cycles and had access to food and water ad libitum. Vivarium temperature was maintained between 22 and 24 °C and humidity was maintained between 50 and 60%.

### 22-[^18^F]FDHA *In Vivo* Uptake and Stability

#### Probe Injection and Tissue Extraction

A modified literature procedure^37^ was performed on female C57BL/6J mice (*n* = 3). Mice anesthetized with isoflurane (1.5–2%) were injected with the 22-[^18^F]FDHA (10.8 ± 1.4 MBq) in formulation through the tail vein. Blood samples were collected from the submandibular vein under anesthesia at 5 and 10 min postinjection (p.i.) and transferred to EDTA-coated microcentrifuge tubes. At 30 min p.i., the mouse was dissected under anesthesia and urine was withdrawn from the bladder. Then, blood was obtained from the inferior vena cava, and the mouse was euthanized by decapitation. The brain was extracted from the skull within 1 min and kept on ice until further processing. Urine was filtered through a 0.22 μm polyvinylidene difluororide filter and analyzed by HPLC (method A, Supporting Information).

#### Folch Extraction

A literature procedure^37^ was performed on plasma (obtained by centrifugation of blood sampled at 30 min p.i.) and brain tissue samples. A 2:1 (v/v) mixture of chloroform/methanol (7 mL) was added at 0 °C to the tissue, which was then homogenized using a hand-held homogenizer. The mixtures were sonicated for 20 s, and 40% aq urea (1.75 mL) and 5% aq H_2_SO_4_ (1.75 mL) were added. After additional sonication for 20 s, the mixtures were centrifuged (1,800 × *g*) for 10 min. Organic, aqueous, and interphase (pellet) fractions were isolated and radioactivity was determined by a gamma counter.

#### *Ex Vivo* Autoradiography

A female mouse was injected with 37 MBq of 22-[^18^F]FDHA in formulation via a tail vein under isoflurane anesthesia (1.5–2%). Another mouse was injected with 41 MBq of [^18^F]fluoride in the same formulation as a control. Mice were kept under maintenance anesthesia for 30 min p.i. and were then euthanized. Blood (*ca*. 0.7 mL) was obtained from the heart and transferred to EDTA-coated microcentrifuge tubes. Each mouse was then perfused with cold PBS and the brain was extracted from the skull. One hemisphere was frozen in cryostat fluid, and 20 μm sagittal sections were then obtained in a cryostat. Frozen brain sections were exposed to a phosphor storage screen (PerkinElmer Super Resolution phosphor screen) overnight. The screen was then read at 600 dpi (42 μm resolution) by using a PerkinElmer Cyclone Plus Storage Phosphor Scanner with OptiQuant software.

### 22-[^18^F]FDHA PET-MR Imaging and Effect of Arecoline

#### Tracer Injection, Arecoline Administration, and PET-MR Imaging

PET-MR image acquisition and reconstruction were performed on a 7T MRI scanner with an integrated PET scanner (MR solutions, Guildford, UK) as described previously,^36^ except that the total scan time was reduced to 32 min (2 min PET baseline acquisition before tracer injection plus 30 min p.i.); list mode data were reconstructed and binned as follows: 4 frames of 30 s, 12 frames of 10 s, 6 frames of 30 s, 5 frames of 60 s, and 10 frames of 120 s.

Six female C57BL/6J mice were divided into a control group and a treatment group (*n* = 3 per group). Each mouse was first injected subcutaneously with saline (control group) or 4 mg/kg methylatropine in saline (treatment group), cannulated at the tail vein, and then placed in the scanner under isoflurane anesthesia (1.5–2%). After 5 min, PET acquisition was started, and after another 2 min, each mouse was injected with 100 μL of formulated 22-[^18^F]FDHA (8.7 ± 1.4 MBq) through the installed cannula. After 10 min, mice were then injected with 100 μL of either saline (control group) or 15 mg/kg arecoline in saline (treatment group) through the same cannula, and image acquisition was continued for an additional 20 min. During PET acquisition, MR images (FSET2w, to define the brain and surrounding area, and respiratory-gated FLASH, to delineate the right ventricle of the heart) were obtained in tandem for defining regions of interest (ROIs). Images were processed using VivoQuant 4.0 (Invicro). PET and MR images were coregistered through the use of a PET/MR phantom. This phantom comprised a capillary tube filled with 10 μL of ∼40 kBq of 22-[^18^F]FDHA in a solution, which was securely positioned on the abdomen of the mouse prior to imaging, serving as a reference point. Three-dimensional regions of interest (ROIs) were delineated through manual segmentation, with the MRI image serving as the anatomical reference. Subsequently, time-activity curves (TAC) were obtained for each mouse within the ROIs corresponding to the right ventricle and the brain.

#### Dynamic PET Kinetic Analysis

For K* estimation, an image-derived input function (IDIF), as a surrogate for the arterial input function, was determined for each mouse as described previously^36,38^ by correcting the TAC of the right ventricle ROI for myocardium spillover. Briefly, the shape of the TACs for each mouse was scaled to that of the venous input function (from measurements of whole blood radioactivity at 5, 10, and 30 min); this scaled IDIF was not corrected for the radiotracer’s metabolites that do not cross the blood-brain barrier. Using the spillover-corrected IDIF and the brain TAC for each mouse, kinetic analysis was performed in MATLAB (version R2022b) using custom numerical fitting routines. First, the spillover-corrected IDIF was fitted into an 8-parameter model, which includes a linear interpolation of the data before the curve’s peak, and the sum of three decreasing exponentials after the peak. Then, kinetic microparameters (*K*_1_, *k*_2_, and *k*_3_) for the irreversible two-tissue compartment model (Irr2TCM) as well as the blood volume fraction in the brain ROI (*V*_B_), were estimated from the brain TAC using the fitted IDIF. The value of the K* in the Irr2TCM, which indicates the net irreversible influx rate of the radiotracer into the brain tissue, was calculated for each set of mouse dynamic PET data from the fitted microparameters following eq 1.
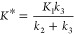
1

## Results and Discussion

### Synthesis of 22-Fluordocosahexaenoic Acid (22-FDHA)

For *in vitro* study of the DHA analogue, we first aimed to synthesize the nonradiolabeled fatty acid, 22-FDHA. We accomplished this starting from the methyl ester of a 22-hydroxylated DHA **10** (Scheme 1), which was prepared by closely following a previously reported method for the modular synthesis of ω-hydroxylated PUFAs.^39^ Briefly, copper-catalyzed coupling of methyl 4-pentynoate and 4-chlorobutyn-1-ol to diyne **1**, followed by bromination to **2**, then another copper-catalyzed alkyne extension into **3**, and a final bromination step afforded triyne **4**. In parallel, the copper-catalyzed coupling of propargyl alcohol and 3-bromo-1-(trimethylsilyl)-1-propyne to **5**, followed by tosylation to **6**, then another copper-catalyzed alkyne extension into **7**, and a final desilylation step afforded triyne **8**. After the copper-catalyzed coupling of **4** and **8**, the hexayne **9** was obtained in a modest yield.

Due to the instability of intermediate **9**, a controlled partial hydrogenation was performed immediately using Lindlar’s catalyst in the presence of 2-methyl-2-butene under H_2_ gas at ambient pressure^39^ to obtain the all-*cis* derivative **10**. The subsequent tosylation step using *p*-toluenesulfonyl chloride under basic anhydrous conditions was performed using crude **10** containing undesired overhydrogenated and incomplete partial-hydrogenated byproducts to obtain crude **11**. However, we successfully isolated the desired tosylate precursor **11** using semipreparative reversed-phase HPLC.

Next, the purified tosylate **11** was subjected to nucleophilic fluorination (Scheme 2).^40^ The reaction was performed according to our previous method for the synthesis of fluorinated AA (20-FAA) using TBAF.^36^ It should be noted that, while the desired fluorinated ester **13** was successfully obtained, only a modest yield (29%) was achieved due to the formation of the 1,2-elimination product **12** (Scheme 2) caused by the intrinsic basicity of TBAF.^41^ Finally, the desired compound, 22-FDHA, was obtained by hydrolyzing ester **13** using LiOH.

### Radiosynthesis of 22-[^18^F]FDHA

**Figure 2:**
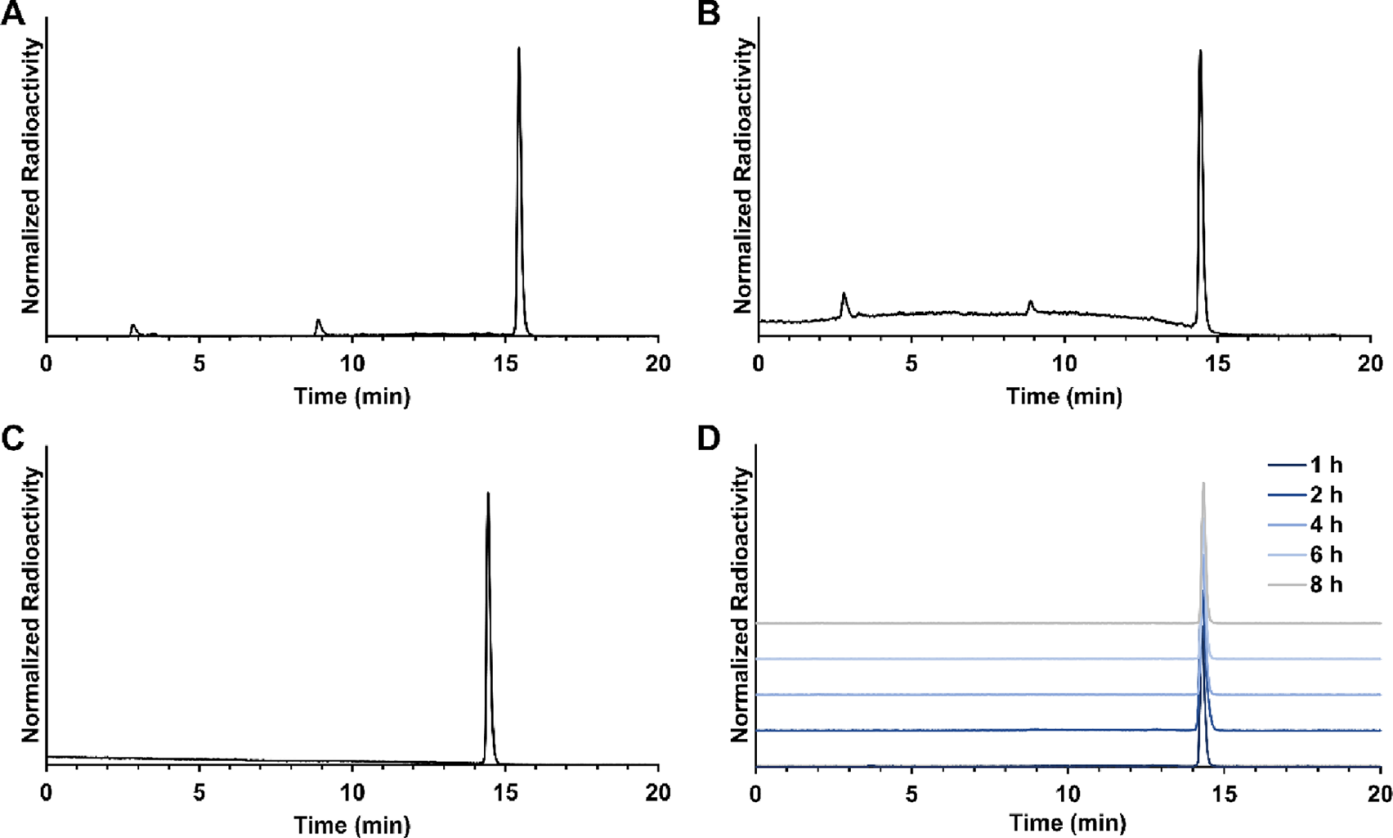
HPLC profiles of (A) crude reaction mixture of methyl 22-[^18^F]fluorodocosahexaenoate ([^18^F]**13**), (B) crude reaction mixture of 22-[^18^F]FDHA, (C) purified 22-[^18^F]FDHA by semipreparative HPLC, and (D) stability of 22-[^18^F]FDHA in mouse serum after incubation for 1, 2, 4, 6, and 8 h at 37 °C.

22-[^18^F]FDHA was successfully prepared through radiofluorination (Scheme 2) with the tosylate **11** by following the procedure for the synthesis of the ^18^F-fluorinated arachidonic acid, 20-[^18^F]FAA.^36^ Briefly, a solution of the tosylate **11** in anhydrous MeCN was treated with a solution of [^18^F]TBAF in MeCN at 85 °C for 20 min, and then the resulting mixture containing the fluorinated ester [^18^F]**13** was treated with 2 M KOH at 85 °C to hydrolyze the intermediate into the free fatty acid. The probe was isolated from the reaction mixture using a single pass semipreparative reverse-phase HPLC, affording 22-[^18^F]FDHA in high radiochemical purity (>99%), with 1.06–6.37% decay-corrected radiochemical yields (Figure 2A–C). The total synthesis time was about 180 min, including the HPLC purification time. Specific activity was 18.0–117.4 GBq/μmol (0.486–3.172 Ci/μmol), which is similar to that previously reported for 20-[^18^F]FAA.^36^

### Log*P* and *In Vitro* Stability

The partition coefficient (log*P*) between 1-octanol and PBS of 22-[^18^F]FDHA was 0.76 ± 0.01. The stability in mouse serum at 37 °C at 1, 2, 4, 6, and 8 h and the percent purities were 98.92 ± 0.01, 98.86 ± 0.01, 98.77 ± 0.03, 98.61 ± 0.29, and 98.33 ± 0.45%, respectively (Figure 2D). After analyzing the stability of the probe in the buffer formulation developed for use in animal imaging (5 mM HEPES in saline containing 50 mg/mL BSA), the percent purity of the probe after 5 h was 95.83 ± 0.23%.

### Bioequivalence Assay

**Figure 3:**
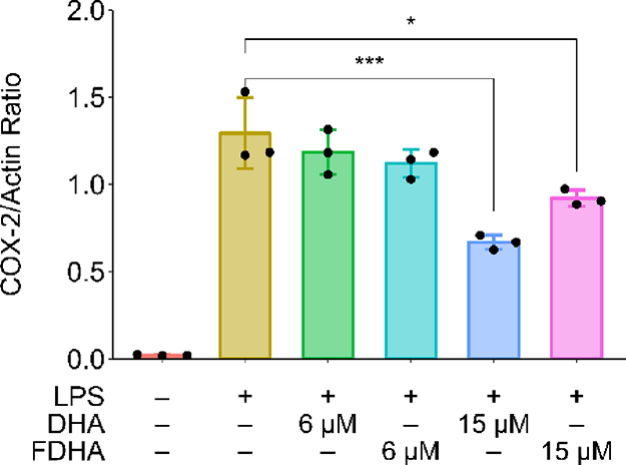
Western blot quantitation of LPS-induced COX-2 induction in BV2 microglial cells, attenuated by treating with DHA and 22-FDHA (*p < 0.05 and ***p < 0.001 by one-way ANOVA with Tukey’s post hoc test).

We evaluated the ability of 22-FDHA to mimic DHA by probing whether fluorination at the ω-position on DHA affects the biochemical role of the natural fatty acid. DHA is a precursor to various specialized pro-resolving mediators and is also anti-inflammatory; it is known to attenuate inflammatory responses of BV-2 microglial cells to LPS.^42,43^ Co-treatment of BV-2 cells with LPS and 6 μM DHA did not show significantly reduced COX-2 expression compared to the LPS-only controls (Figure 3). However, a significant reduction was observed for both DHA and 22-FDHA at 15 μM with no significant difference in COX-2 induction in LPS-treated microglia cotreated with either compound (*p* > 0.05). Therefore, it is likely that fluorination at the ω-position on DHA can preserve the ability of DHA to be recognized, transported, and metabolized in the brain, making 22-[^18^F]FDHA a suitable probe for monitoring DHA uptake *in vivo*.

### *In Vivo* Uptake and Stability

To assess the nature of the radioactive species contributing to the activity in tissues, as a measure of the *in vivo* stability of 22-[^18^F]FDHA, Folch extraction was performed. In this assay, [^18^F]fluoride ions and low molecular-weight metabolites were extracted into the hydrophilic phase and 22-[^18^F]FDHA and lipid metabolites were extracted into the hydrophobic phase. Radioactivity partitioned into organic, aqueous, and interphase (pellet) fractions of blood and brain tissue obtained from mice injected with 22-[^18^F]FDHA was detected as shown in Table 1. A significant amount of radioactivity was detected in the hydrophilic phase obtained from both blood and brain samples. As observed on radio HPLC (method A, Supporting Information), two radioactive compounds were present in the urine collected 30 min p.i. The peak at 3.1 min, which constitutes approximately 60% of the radioactivity, corresponds to [^18^F]fluoride ions. The remaining 40% of the radioactivity was detected from the peak that appeared at 6.8 min, which likely corresponds to a metabolite that is significantly more hydrophilic than 22-[^18^F]FDHA. No peak corresponding to the parent probe 22-[^18^F]FDHA was present (data not shown) as we observed with 20-[^18^F]FAA, indicating that the probe had been cleared as hydrophilic metabolites.^28^

**Table 1:** Radioactivity in Fractions Obtained from Folch Extraction in Blood, Brain Tissues, and Urine of Mice (*n* = 3) 30 min after i.v. Administration of 22-[^18^F]FDHA^a^

		**Folch extraction**		
**tissue**	**%ID/g**	organic	aqueous	interphase
blood	5.2 ± 1.3	61.5 ± 5.5%	33.5 ± 5.4%	4.9 ± 0.6%
brain	1.10 ± 0.2	58.0 ± 2.2%	32.3 ± 3.0%	9.6 ± 1.1%
urine	19.3 ± 2.9			

a Mean ± SD (*n* = 3).

### *Ex Vivo* Analysis

**Figure 4:**
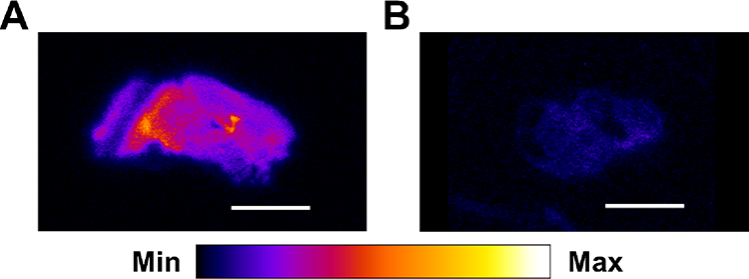
*Ex vivo* brain autoradiography of mice 30 min after injection of (A) 22-[^18^F]FDHA (37 MBq) or (B) sodium [^18^F]fluoride (41 MBq). Scale bar: 5 mm.

We have confirmed through autoradiography (Figure 4) that activity can be detected in the brain tissue of a mouse 30 min p.i. with 22-[^18^F]FDHA. The uptake and distribution are distinct from those of the brain of another mouse injected with a similar amount of sodium [^18^F]fluoride in the same formulation. This suggests that the tracer is taken up and accreted into the brain within 30 min.

In addition, we conducted an *in vivo* uptake study by administering nonradioactive 22-FDHA via intravenous injection into C57BL/6 mice. After tissue extraction from plasma, brain, and liver, we employed multiple reaction monitoring via LC-MS (Supporting Information) to successfully detect free 22-FDHA in these tissues (Figures S1–S3, Supporting Information). Following a single administration of 22-FDHA, the compound was detectable in both the plasma and brain after 30 min. Notably, a dose-dependent increase was observed in both compartments (Table 2), with plasma levels reaching 10 ng/mL and brain levels reaching 1 ng/g for the mouse receiving a 1 μg dose. Furthermore, the administration of a higher dosage of 22-FDHA resulted in a larger fraction being taken up by the brain, as evidenced by the elevated brain/plasma ratio (10.5% vs 5.6%) for the 1 μg dose compared to the 0.5 μg dose.

### PET-MR Imaging of 22-[^18^F]FDHA Brain Uptake *In Vivo*

**Figure 5:**
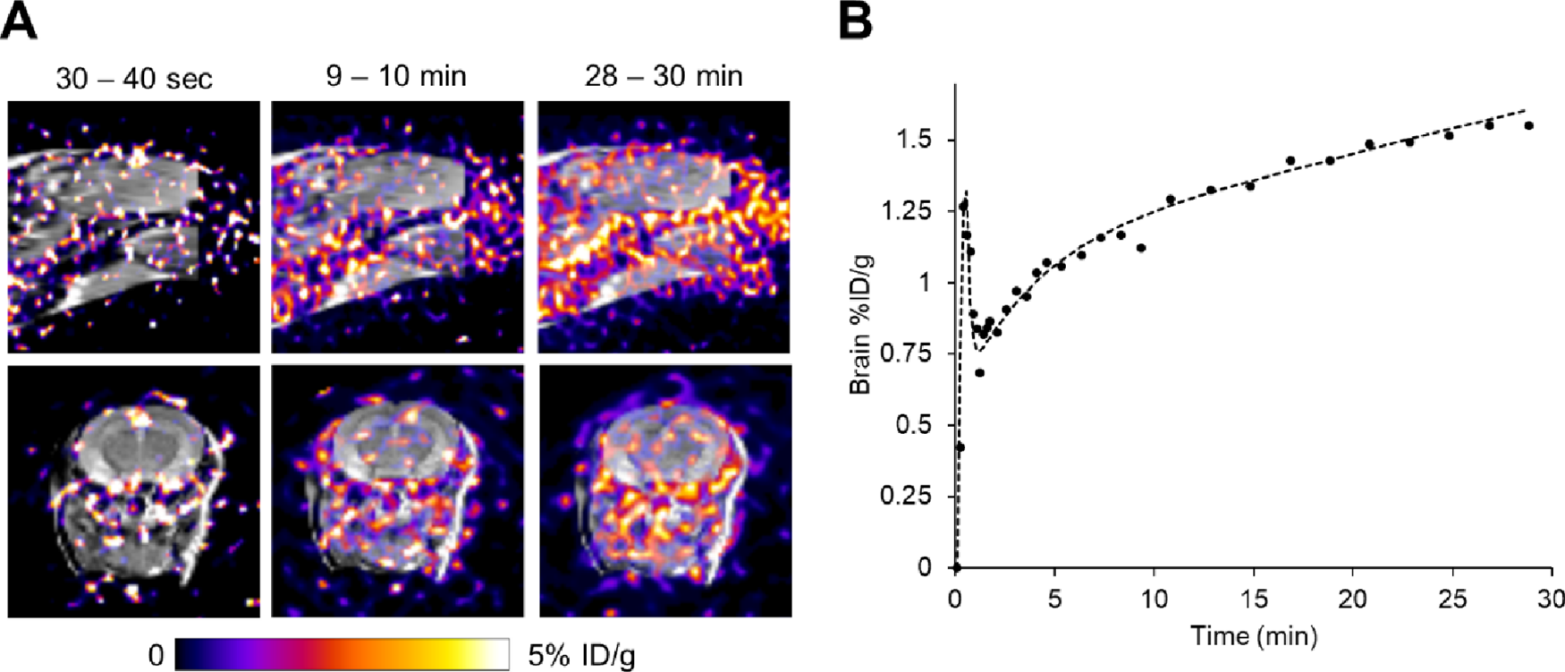
(A) Superimposed PET-MR images of a representative mouse brain at different time points. (B) Time–activity curve of a representative mouse after i.v. injection of 22-[^18^F]FDHA.

Like 20-[^18^F]FAA,^36^ ROI analysis of the dynamic PET images using 22-[^18^F]FDHA showed that the probe was mostly taken up in nonbrain regions such as the liver and kidneys, with only about 0.5% of the total probe [ca. 1.4% injected dose per gram (%ID/g), Figure 5A] entering the mouse brain after 30 min. In the brain ROI, we observed for 22-[^18^F]FDHA a pattern of a sharp increase followed by a slow, steady increase in radioactivity (Figure 5B). In our PET imaging experiments using normal nude mice, marginal increases in radioactivity were found in brain ROI at 60 min p.i. (*ca*. 1.6%ID/g, Figure S4, Supporting Information).

### Effect of Arecoline

**Figure 6:**
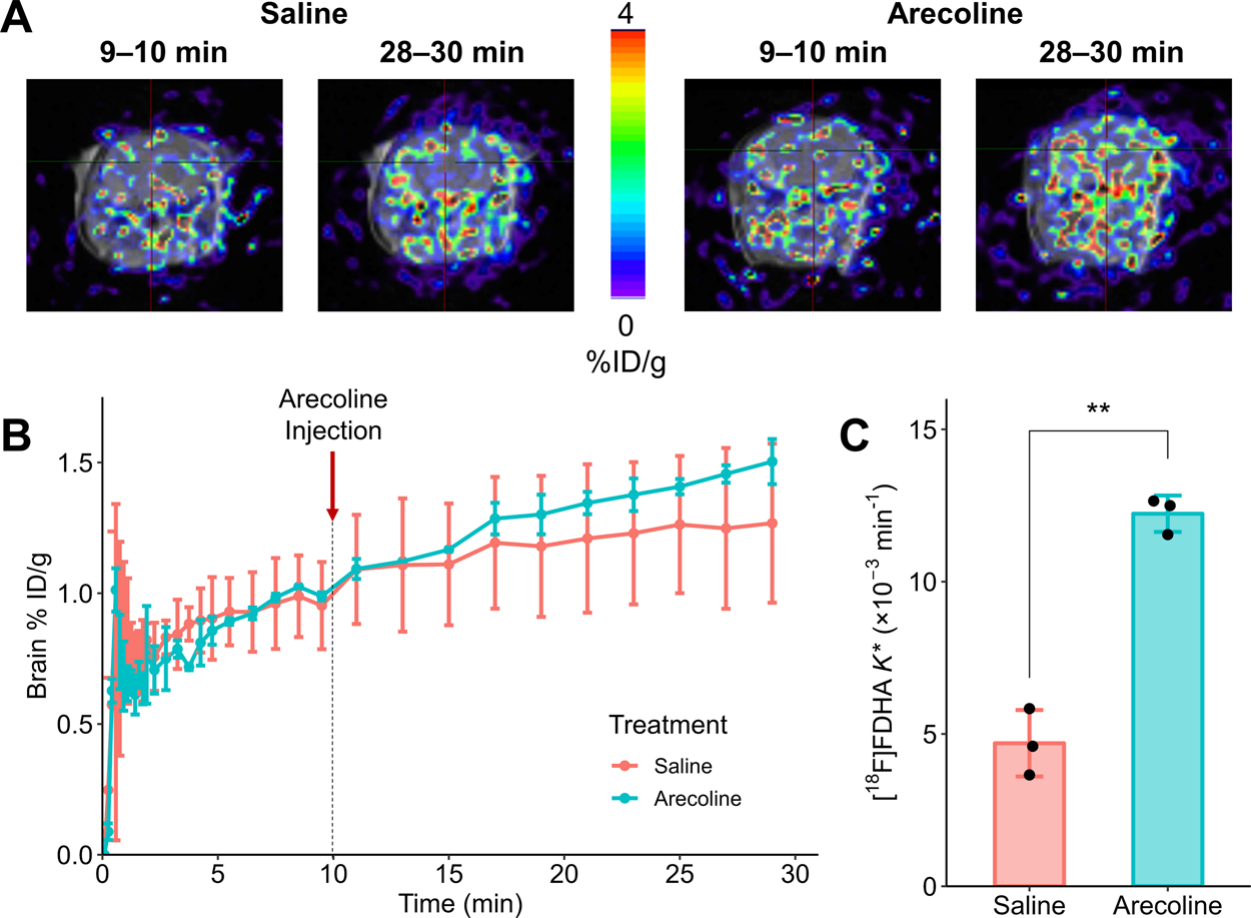
(A) PET-MR images at the 9–10 and 28–30 min frames for representative mice treated with saline or arecoline. (B) %ID/g over time for mice treated with saline or 15 mg/kg arecoline (*n* = 3/group). The mean %ID/g values measured for mice treated with arecoline were not significantly higher than those of control mice. (C) Incorporation coefficients (K*) estimated by Irr2TCM using each mouse’s TAC as the output function. 22-[^18^F]FDHA K* estimates for mice treated with arecoline were significantly higher (**p < 0.01, Student’s *t*-test) than control mice.

Arecoline, a muscarinic acetylcholine receptor agonist, has been shown to increase K* for DHA (measured by autoradiography) in rodents through the activation of calcium-independent phospholipase A2 (iPLA_2_).^23,24,27^ DHA is preferentially cleaved from the 2-position of phospholipids by iPLA, and the activation of the enzyme is known to affect DHA cycling in the brain.^24,44^ Thus, we investigated the effect of arecoline administration on the K* estimate by 22-[^18^F]FDHA dynamic PET. Mice were first pretreated subcutaneously with methylatropine (4 mg/kg), to reduce systemic effects without affecting K*,^27^ and then intravenously injected with a bolus of 22-[^18^F]FDHA. During PET acquisition, mice were then intravenously injected with saline or arecoline in saline (15 mg/kg) at 10 min p.i. of the probe. The %ID/g measured in the brain ROI at the last time point (28–30 min) was not significantly different between control and arecoline-treated mice (Figure 6A,B). However, there was an apparent increase in the rate of uptake (which could be revealed by K* estimation) into the brains of mice after iv injection of arecoline.

Through kinetic modeling of the dynamic PET data, we found a significantly higher mean DHA K* estimate in mice injected with arecoline (Figure 6C and Table 3, 160% increase of mean K* value; *p* = 0.0016 by Student’s *t* test); increases ranging between 68 and 120% for rodents subcutaneously injected with arecoline were previously reported (Table 3).^23,24,27^K* estimates for the control mice studied were much lower than those determined in unanesthetized mice by autoradiography using 1-[^14^C]DHA^24^ but comparable to values found for rats^23,27^ and to values estimated in humans by PET (Table 3).^33,34^ The lower values are attributed to the fact that we did not account for the metabolite fraction in the whole blood samples used to correct the IDIFs, and radioactivity in whole blood does not always equate radioactivity in plasma, which is a factor in the “scaling” of the K* estimates. In addition, the uptake of fatty acids was monitored in anesthetized mice.

**Table 2:** 22-FDHA Concentrations in Tissues at 30 min after i.v. Injection

	**22-FDHA concentration**			**22-FDHA tissue/plasma ratio**	
	**plasma (ng/mL)**	**brain (ng/g)**	**liver (ng/g)**	**brain/plasma**	**liver/plasma**
mouse 1 (1 μg)	10.16	1.07	0.28	10.5%	2.8%
mouse 2 (0.5 μg)	3.60	0.20	0.16	5.6%	4.4%

**Table 3:** Estimates of the Brain Incorporation Coefficient (K*)

			K* (×10^**–3**^**min**^**–1**^**)**		
**methods**	**DHA probes**	**models**	**control**	**arecoline**	**ref**
autoradiography	4,5-[^3^H]DHA	rats	1.9	3.3	(23)
	1-[^14^C]DHA	mice	79.9–258.4	175.5–494.9	(24)
	1-[^14^C]DHA	rats	13.9	23.3	(27)
PET-MR	1-[^11^C]DHA	humans	4.0		(33)^,^(34)
	22-[^18^F]FDHA	mice	4.69 ± 1.09^a^	12.20 ± 0.60^a^	this work

a Mean ± SD (*n* = 3).

## Conclusions

We have successfully developed, synthesized, and characterized a PET probe, 22-[^18^F]FDHA, designed for visualizing DHA K* in the brains of mice. In our cellular studies using the nonradiolabeled 22-FDHA, we observed that fluorination did not impact the biological activity of DHA *in vitro*. Following a protocol that we have previously employed for PET-MR imaging with 20-[^18^F]FAA in mice, we were able to accurately estimate K* through dynamic PET-MR imaging with 22-[^18^F]FDHA. Moreover, we observed the anticipated increase in the K* value in mice treated with arecoline. Upon successful translation and validation in human subjects, 22-[^18^F]DHA PET holds the potential to serve multiple important purposes, including (1) identifying factors that influence brain DHA homeostasis, such as APOE4, aging, exercise, iPLA2 activity, and the presence of AD pathology; (2) assisting in the selection of study participants based on their brain DHA uptake kinetics; and (3) guiding interventions aimed at improving the delivery of DHA to the brain, which could be explored as a preventive or therapeutic strategy.

## Supporting Information Available

Details on general chemistry information, HPLC methods, synthetic procedures, NMR spectra, LC-MS analysis, and PET imaging data (PDF)

## References

[ref1] Weiser M. J.; Butt C. M.; Mohajeri M. H. Docosahexaenoic Acid and Cognition throughout the Lifespan. Nutrients 2016, 8 (2), 99 10.3390/nu8020099. 26901223 PMC4772061

[ref2] Lacombe R. J. S.; Chouinard-Watkins R.; Bazinet R. P. Brain Docosahexaenoic Acid Uptake and Metabolism. Mol. Aspects Med. 2018, 64, 109–134. 10.1016/j.mam.2017.12.004. 29305120

[ref3] Calder P. C. Very Long-Chain N-3 Fatty Acids and Human Health: Fact, Fiction and the Future. Proc. Nutr. Soc. 2018, 77 (1), 52–72. 10.1017/S0029665117003950. 29039280

[ref4] Rapoport S. I. In Vivo Fatty Acid Incorporation into Brain Phosholipids in Relation to Plasma Availability, Signal Transduction and Membrane Remodeling. J. Mol. Neurosci. 2001, 16 (2), 243–261. 10.1385/JMN:16:2-3:243. 11478380

[ref5] Demar J. C. Jr.; Ma K.; Chang L.; Bell J. M.; Rapoport S. I. α-Linolenic Acid Does Not Contribute Appreciably to Docosahexaenoic Acid within Brain Phospholipids of Adult Rats Fed a Diet Enriched in Docosahexaenoic Acid. J. Neurochem. 2005, 94 (4), 1063–1076. 10.1111/j.1471-4159.2005.03258.x. 16092947

[ref6] Chen C. T.; Kitson A. P.; Hopperton K. E.; Domenichiello A. F.; Trepanier M. O.; Lin L. E.; Ermini L.; Post M.; Thies F.; Bazinet R. P. Plasma Non-esterified Docosahexaenoic Acid is the Major Pool Supplying the Brain. Sci. Rep. 2015, 5, 15791. 26511533 10.1038/srep15791 PMC4625162

[ref7] Cunnane S. C.; Schneider J. A.; Tangney C.; Tremblay-Mercier J.; Fortier M.; Bennett D. A.; Morris M. C. Plasma and Brain Fatty Acid Profiles in Mild Cognitive Impairment and Alzheimer’s Disease. J. Alzheimers Dis. 2012, 29 (3), 691–697. 10.3233/JAD-2012-110629. 22466064 PMC3409580

[ref8] Cunnane S. C.; Chouinard-Watkins R.; Castellano C. A.; Barberger-Gateau P. Docosahexaenoic Acid Homeostasis, Brain Aging and Alzheimer’s Disease: Can We Reconcile the Evidence?. Prostaglandins Leukot. Essent. Fatty Acids 2013, 88 (1), 61–70. 10.1016/j.plefa.2012.04.006. 22575581

[ref9] Liu C. C.; Liu C. C.; Kanekiyo T.; Xu H.; Bu G. Apolipoprotein E and Alzheimer Disease: Risk. Mechanisms and Therapy. Nat. Rev. Neurol. 2013, 9 (2), 106–118. 23296339 10.1038/nrneurol.2012.263 PMC3726719

[ref10] Vandal M.; Alata W.; Tremblay C.; Rioux-Perreault C.; Salem N. Jr.; Calon F.; Plourde M. Reduction in DHA Transport to the Brain of Mice Expressing Human APOE4 Compared to APOE2. J. Neurochem. 2014, 129 (3), 516–526. 10.1111/jnc.12640. 24345162

[ref11] Pontifex M. G.; Martinsen A.; Saleh R. N. M.; Harden G.; Tejera N.; Muller M.; Fox C.; Vauzour D.; Minihane A. M. APOE4 Genotype Exacerbates the Impact of Menopause on Cognition and Synaptic Plasticity in APOE-TR Mice. FASEB J. 2021, 35 (5), e21583. 33891334 10.1096/fj.202002621RR

[ref12] Duro M. V.; Ebright B.; Yassine H. N. Lipids and Brain Inflammation in APOE4-Associated Dementia. Curr. Opin. Lipidol. 2022, 33 (1), 16–24. 10.1097/MOL.0000000000000801. 34907965 PMC8769806

[ref13] Ebright B.; Assante I.; Poblete R. A.; Wang S.; Duro M. V.; Bennett D. A.; Arvanitakis Z.; Louie S. G.; Yassine H. N. Eicosanoid Lipidome Activation in Post-Mortem Brain Tissues of Individuals with APOE4 and Alzheimer’s Dementia. Alzheimers Res. Ther. 2022, 14 (1), 152. 36217192 10.1186/s13195-022-01084-7 PMC9552454

[ref14] Chouinard-Watkins R.; Rioux-Perreault C.; Fortier M.; Tremblay-Mercier J.; Zhang Y.; Lawrence P.; Vohl M. C.; Perron P.; Lorrain D.; Brenna J. T.; et al. Disturbance in Uniformly ^13^C-Labelled DHA Metabolism in Elderly Human Subjects Carrying the ApoE Epsilon4 Allele. Br. J. Nutr. 2013, 110 (10), 1751–1759. 10.1017/S0007114513001268. 23631810

[ref15] Tomaszewski N.; He X.; Solomon V.; Lee M.; Mack W. J.; Quinn J. F.; Braskie M. N.; Yassine H. N. Effect of APOE Genotype on Plasma Docosahexaenoic Acid (DHA), Eicosapentaenoic Acid, Arachidonic Acid, and Hippocampal Volume in the Alzheimer’s Disease Cooperative Study-Sponsored DHA Clinical Trial. J. Alzheimers Dis. 2020, 74 (3), 975–990. 10.3233/JAD-191017. 32116250 PMC7156328

[ref16] Coughlan G.; Larsen R.; Kim M.; White D.; Gillings R.; Irvine M.; Scholey A.; Cohen N.; Legido-Quigley C.; Hornberger M.; et al. APOE Epsilon4 Alters Associations Between Docosahexaenoic Acid and Preclinical Markers of Alzheimer’s Disease. Brain Commun. 2021, 3 (2), fcab085. 34007965 10.1093/braincomms/fcab085 PMC8112902

[ref17] Bantugan M. A.; Xian H.; Solomon V.; Lee M.; Cai Z.; Wang S.; Duro M. V.; Kerman B. E.; Fonteh A.; Meuret C.; et al. Associations of ApoE4 Status and DHA Supplementation on Plasma and CSF Lipid Profiles and Entorhinal Cortex Thickness. J. Lipid Res. 2023, 64 (6), 100354 10.1016/j.jlr.2023.100354. 36958720 PMC10230261

[ref18] Arellanes I. C.; Choe N.; Solomon V.; He X.; Kavin B.; Martinez A. E.; Kono N.; Buennagel D. P.; Hazra N.; Kim G.; et al. Brain Delivery of Supplemental Docosahexaenoic Acid (DHA): A Randomized Placebo-Controlled Clinical Trial. EBioMedicine 2020, 59. 10.1016/j.ebiom.2020.102883 PMC7502665 32690472

[ref19] Yassine H. N. Targeting Prodromal Alzheimer’s Disease: Too Late for Prevention?. Lancet Neurol. 2017, 16 (12), 946–947. 10.1016/S1474-4422(17)30372-1. 29097165 PMC5808883

[ref20] Balakrishnan J.; Husain M. A.; Vachon A.; Chouinard-Watkins R.; Léveillé P.; Plourde M. Omega-3 Supplementation Increases Omega-3 Fatty Acids in Lipid Compartments That Can Be Taken Up by the Brain Independent of APOE Genotype Status: A Secondary Analysis from A Randomised Controlled Trial. Nutr. Healthy Aging 2022, 7 (3–4), 147–158. 10.3233/NHA-220169.

[ref21] Husain M. A.; Vachon A.; Chouinard-Watkins R.; Vandal M.; Calon F.; Plourde M. Investigating the Plasma-Liver-Brain Axis of Omega-3 Fatty Acid Metabolism in Mouse Knock-In for the Human Apolipoprotein E Epsilon 4 Allele. J. Nutr. Biochem. 2023, 111, 109181 10.1016/j.jnutbio.2022.109181. 36220526

[ref22] Wang S.; Li B.; Solomon V.; Fonteh A.; Rapoport S. I.; Bennett D. A.; Arvanitakis Z.; Chui H. C.; Sullivan P. M.; Yassine H. N. Calcium-Dependent Cytosolic Phospholipase A2 Activation Is Implicated in Neuroinflammation and Oxidative Stress Associated with ApoE4. Mol. Neurodegener. 2022, 17 (1), 42. 35705959 10.1186/s13024-022-00549-5 PMC9202185

[ref23] Jones C. R.; Arai T.; Rapoport S. I. Evidence for the Involvement of Docosahexaenoic Acid in Cholinergic Stimulated Signal Transduction at the Synapse. Neurochem. Res. 1997, 22 (6), 663–670. 10.1023/A:1027341707837. 9178948

[ref24] Basselin M.; Rosa A. O.; Ramadan E.; Cheon Y.; Chang L.; Chen M.; Greenstein D.; Wohltmann M.; Turk J.; Rapoport S. I. Imaging Decreased Brain Docosahexaenoic Acid Metabolism and Signaling in iPLA_2_β (VIA)-Deficient Mice. J. Lipid Res. 2010, 51 (11), 3166–3173. 10.1194/jlr.M008334. 20686114 PMC2952557

[ref25] Basselin M.; Ramadan E.; Rapoport S. I. Imaging Brain Signal Transduction and Metabolism via Arachidonic and Docosahexaenoic Acid in Animals and Humans. Brain Res. Bull. 2012, 87 (2–3), 154–171. 10.1016/j.brainresbull.2011.12.001. 22178644 PMC3274571

[ref26] Igarashi M.; Kim H. W.; Chang L.; Ma K.; Rapoport S. I. Dietary n-6 Polyunsaturated Fatty Acid Deprivation Increases Docosahexaenoic Acid Metabolism in Rat Brain. J. Neurochem. 2012, 120 (6), 985–997. 10.1111/j.1471-4159.2011.07597.x. 22117540 PMC3296886

[ref27] DeGeorge J. J.; Nariai T.; Yamazaki S.; Williams W. M.; Rapoport S. I. Arecoline-Stimulated Brain Incorporation of Intravenously Administered Fatty Acids in Unanesthetized Rats. J. Neurochem. 1991, 56 (1), 352–355. 10.1111/j.1471-4159.1991.tb02603.x. 1824784

[ref28] Lacombe R. J. S.; Lee C. C.; Bazinet R. P. Turnover of Brain DHA in Mice is Accurately Determined by Tracer-Free Natural Abundance Carbon Isotope Ratio Analysis. J. Lipid Res. 2020, 61 (1), 116–126. 10.1194/jlr.D119000518. 31712249 PMC6939594

[ref29] Yoshinaga K.; Usami Y.; Yoshinaga-Kiriake A.; Shikano H.; Taira S.; Nagasaka R.; Tanaka S.; Gotoh N. Visualization of Dietary Docosahexaenoic Acid in Whole-Body Zebrafish Using Matrix-Assisted Laser Desorption/Ionization Mass Spectrometry Imaging. J. Nutr. Biochem. 2022, 100, 108897 10.1016/j.jnutbio.2021.108897. 34748923

[ref30] Yoshinaga K.; Ishikawa H.; Taira S.; Yoshinaga-Kiriake A.; Usami Y.; Gotoh N. Selective Visualization of Administrated Arachidonic and Docosahexaenoic Acids in Brain Using Combination of Simple Stable Isotope-Labeling Technique and Imaging Mass Spectrometry. Anal. Chem. 2020, 92 (13), 8685–8690. 10.1021/acs.analchem.0c01289. 32468807

[ref31] Plourde M.; Chouinard-Watkins R.; Vandal M.; Zhang Y.; Lawrence P.; Brenna J. T.; Cunnane S. C. Plasma Incorporation, Apparent Retroconversion and Beta-Oxidation of ^13^C-Docosahexaenoic Acid in the Elderly. Nutr. Metab. (Lond) 2011, 8, 5 10.1186/1743-7075-8-5. 21272363 PMC3038891

[ref32] Umhau J. C.; Zhou W.; Carson R. E.; Rapoport S. I.; Polozova A.; Demar J.; Hussein N.; Bhattacharjee A. K.; Ma K.; Esposito G.; et al. Imaging Incorporation of Circulating Docosahexaenoic Acid into the Human Brain Using Positron Emission Tomography. J. Lipid Res. 2009, 50 (7), 1259–1268. 10.1194/jlr.M800530-JLR200. 19112173 PMC2694326

[ref33] Umhau J. C.; Zhou W.; Thada S.; Demar J.; Hussein N.; Bhattacharjee A. K.; Ma K.; Majchrzak-Hong S.; Herscovitch P.; Salem N. Jr.; et al. Brain Docosahexaenoic Acid [DHA] Incorporation and Blood Flow are Increased in Chronic Alcoholics: A Positron Emission Tomography Study Corrected for Cerebral Atrophy. PLoS One 2013, 8 (10), e75333 10.1371/journal.pone.0075333. 24098376 PMC3788756

[ref34] Yassine H. N.; Croteau E.; Rawat V.; Hibbeln J. R.; Rapoport S. I.; Cunnane S. C.; Umhau J. C. DHA Brain Uptake and APOE4 Status: A PET Study with [1-^11^C]-DHA. Alzheimers Res. Ther. 2017, 9 (1), 23. 28335828 10.1186/s13195-017-0250-1 PMC5364667

[ref35] Ali S.; Zhou J. Highlights on U.S. FDA-Approved Fluorinated Drugs Over the Past Five Years (2018–2022). Eur. J. Med. Chem. 2023, 256, 115476 10.1016/j.ejmech.2023.115476. 37207534 PMC10247436

[ref36] Van Valkenburgh J.; Duro M. V. V.; Burnham E.; Chen Q.; Wang S.; Tran J.; Kerman B. E.; Hwang S. H.; Liu X.; Sta Maria N. S.; et al. Radiosynthesis of 20-[^18^F]Fluoroarachidonic Acid for PET-MR Imaging: Biological Evaluation in ApoE4-TR Mice. Prostaglandins Leukot. Essent. Fatty Acids 2022, 186, 102510. 36341886 10.1016/j.plefa.2022.102510 PMC9888757

[ref37] DeGrado T. R.; Bhattacharyya F.; Pandey M. K.; Belanger A. P.; Wang S. Synthesis and Preliminary Evaluation of ^18^F-Fluoro-4-Thia-Oleate as a PET Probe of Fatty Acid Oxidation. J. Nucl. Med. 2010, 51 (8), 1310 10.2967/jnumed.109.074245. 20660391

[ref38] Zhou S.; Chen K.; Reiman E. M.; Li D. M.; Shan B. A Method for Generating Image-Derived Input Function in Quantitative ^18^F-FDG PET Study Based on the Monotonicity of the Input and Output Function Curve. Nucl. Med. Commun. 2012, 33 (4), 362–370. 10.1097/MNM.0b013e32834f262e. 22262245 PMC3654793

[ref39] Hwang S. H.; Wagner K.; Xu J.; Yang J.; Li X.; Cao Z.; Morisseau C.; Lee K. S.; Hammock B. D. Chemical Synthesis and Biological Evaluation of ω-Hydroxy Polyunsaturated Fatty Acids. Bioorg. Med. Chem. Lett. 2017, 27 (3), 620–625. 10.1016/j.bmcl.2016.12.002. 28025003 PMC5584617

[ref40] Tungen J. E.; Aursnes M.; Ramon S.; Colas R. A.; Serhan C. N.; Olberg D. E.; Nuruddin S.; Willoch F.; Hansen T. V. Synthesis of Protectin D1 Analogs: Novel Pro-resolution and Radiotracer Agents. Org. Biomol. Chem. 2018, 16 (36), 6818–6823. 10.1039/C8OB01232F. 30204204 PMC6309860

[ref41] Sun H.; DiMagno S. G. TBAF Fluorination for Preparing Alkyl Fluorides. In Fluorination 2018, 1–10.

[ref42] Yang B.; Li R.; Michael Greenlief C.; Fritsche K. L.; Gu Z.; Cui J.; Lee J. C.; Beversdorf D. Q.; Sun G. Y. Unveiling Anti-Oxidative and Anti-Inflammatory Effects of Docosahexaenoic Acid and Its Lipid Peroxidation Product on Lipopolysaccharide-Stimulated BV-2 Microglial Cells. J. Neuroinflammation 2018, 15 (1), 202. 29986724 10.1186/s12974-018-1232-3 PMC6038194

[ref43] Lu D. Y.; Tsao Y. Y.; Leung Y. M.; Su K. P. Docosahexaenoic Acid Suppresses Neuroinflammatory Responses and Induces Heme Oxygenase-1 Expression in BV-2 Microglia: Implications of Antidepressant Effects for Omega-3 Fatty Acids. Neuropsychopharmacology 2010, 35 (11), 2238–2248. 10.1038/npp.2010.98. 20668435 PMC3055314

[ref44] Rosa A. O.; Rapoport S. I. Intracellular- and Extracellular-Derived Ca^2+^ Influence Phospholipase A_2_-Mediated Fatty Acid Release from Brain Phospholipids. Biochim. Biophys. Acta 2009, 1791 (8), 697–705. 10.1016/j.bbalip.2009.03.009. 19327408 PMC2735787

