# Supplementary material for: Synthesis and Preclinical Evaluation of 22-[18F]Fluorodocosahexaenoic Acid as a Positron Emission Tomography Probe for Monitoring Brain Docosahexaenoic Acid Uptake Kinetics
Source: ACS Chem Neurosci. 2023 Dec 4;14(24):4409–18. doi: 10.1021/acschemneuro.3c00681 (PMC10739598; doi:10.1021/acschemneuro.3c00681)
Supplement: Supplementary file 1 — cn3c00681_si_001.pdf [file cn3c00681_si_001.pdf]

Supporting Information

Synthesis and Preclinical Evaluation of 22-[^{18}F]Fluorodocosahexaenoic Acid as a Positron Emission Tomography Probe for Monitoring Brain Docosahexaenoic Acid Uptake Kinetics

Marlon Vincent V. Duro,[1,*] Juno Van Valkenburgh,[1,*] Diana E. Ingles,[2] Jenny Tran,[2] Zhiheng Cai,[2] Brandon Ebright,[3] Shaowei Wang,[2] Bilal E. Kerman,[2] Jasmin Galvan,[2] Sung Hee Hwang,[4] Naomi S. Sta Maria,[5] Francesca Zanderigo,[6,7] Etienne Croteau,[8] Stephen C. Cunnane,[9] Stanley I. Rapoport,[10] Stan G. Louie,[3] Russell E. Jacobs,[5] Hussein N. Yassine,[2,**] Kai Chen[1,**]

[1]Department of Radiology, Keck School of Medicine, University of Southern California, Los Angeles, CA 90033, United States

[2]Department of Medicine, Keck School of Medicine, University of Southern California, Los Angeles, CA 90033, United States

[3]Alfred E. Mann School of Pharmacy and Pharmaceutical Sciences, University of Southern California, Los Angeles, CA 90089, United States

[4]Department of Entomology and Nematology and UC Davis Comprehensive Cancer Center, University of California, Davis, CA 95616, United States

[5]Zilkha Neurogenetic Institute, Keck School of Medicine, University of Southern California, Los Angeles, CA 90033, United States

[6]Department of Psychiatry, Columbia University, New York, NY 10032, United States

[7]Molecular Imaging and Neuropathology Area, New York State Psychiatric Institute, New York, NY 10032, United States

[8]Sherbrooke Center for Molecular Imaging, University of Sherbrooke Sherbrooke, QC, J1H 4C4, Canada

[9]Research Center on Aging, Department of Medicine, University of Sherbrooke, Sherbrooke, QC, J1H 4C4, Canada

[10]National Institute on Alcohol Abuse and Alcoholism, Bethesda, MD, United States

* Equal contributions

**Co-senior authors with equal contributions

Correspondence:

Hussein N. Yassine, hyassine@usc.edu

Kai Chen, chenkai@med.usc.edu

TABLE OF CONTENTS

General Chemistry 3

HPLC Methods 4

- Method A (Analytical HPLC) 4
- Method B (Semi-preparative HPLC) 4
- Method C (Semi-preparative HPLC) 4

Synthetic Procedures 5

- Methyl 9-Hydroxynona-4,7-diynoate (**1**) 5
- Methyl 9-Bromonona-4,7-diynoate (**2**) 5
- Methyl 12-Hydroxydodeca-4,7,10-triynoate (**3**) 6
- Methyl 12-Bromododeca-4,7,10-triynoate (**4**) 6
- 6-(Trimethylsilyl)hexa-2,5-diyn-1-ol (**5**) 7
- 6-(Trimethylsilyl)hexa-2,5-diyn-1-yl 4-methylbenzenesulfonate (**6**) 7
- 10-(Trimethylsilyl)deca-3,6,9-triyn-1-ol (**7**) 8
- Deca-3,6,9-triyn-1-ol (**8**) 8
- Methyl (4*Z*,7*Z*,10*Z*,13*Z*,16*Z*,19*Z*)-22-(Tosyloxy)docosa-4,7,10,13,16,19-hexaenoate (**11**) 9
  - Methyl 22-Hydroxydocosa-4,7,10,13,16,19-hexaynoate (**9**) 10
  - Methyl (4Z,7Z,10Z,13Z,16Z,19Z)-22-Hydroxydocosa-4,7,10,13,16,19-hexaenoate (**10**) 11
- Methyl (4*Z*,7*Z*,10*Z*,13*Z*,16*Z*,19*Z*)-22-Fluorodocosa-4,7,10,13,16,19-hexaenoate (**13**) 11
  - Methyl (4Z,7Z,10Z,13Z,16Z,19Z)-Docosa-4,7,10,13,16,19,21-heptaenoate (**12**) 11
- (4*Z*,7*Z*,10*Z*,13*Z*,16*Z*,19*Z*)-22-Fluorodocosa-4,7,10,13,16,19-hexaenoic acid (22-FDHA) 12

NMR Spectra 13

Ex Vivo LC-MS Analysis of 22-FDHA 25

PET Imaging of 22-[^{18}F]FDHA in Normal Nude Mice 30

REFERENCES 30

General Chemistry

All reagents and solvents were obtained from various commercial sources and used without further purification unless otherwise noted. All reactions were performed under nitrogen gas unless otherwise mentioned. Thin-layer chromatography (TLC) was performed on precoated silica gel 60 Å plates with UV 254 indicator and visualized with either UV light or a $KMnO_4$ stain. Automated flash chromatography was performed using a Teledyne ISCO Combi*Flash* Rf 200 automatic chromatography system with Redi*Sep* prepacked gel cartridges. Nuclear magnetic resonance (NMR) spectra were recorded on a 400 MHz Oxford NMR AS400, a 400 MHz Varian Mercury, or a 500 MHz Varian VNMRS NMR spectrometer. Proton and ^{13}C NMR chemical shifts are reported in ppm (δ) downfield from TMS using internal TMS or residual protonated solvent as reference ($CHCl_3$: 7.26 ppm for ^{1}H and 77.16 ppm for ^{13}C). Fluorine-19 NMR chemical shifts are reported in ppm downfield from $CFCl_3$ using the deuterium lock channel as a reference. NMR data are reported as follows: chemical shift, multiplicity (s = singlet, d = doublet, t = triplet, q = quartet, p = pentet, dt = triplet of doublets, dq = quartet of doublets, tt = triplet of triplets, m = multiplet), coupling constant (*J* in Hz), and integration. Low-resolution mass spectra were obtained on an MSQ Plus single quadrupole mass spectrometer in positive mode with an electrospray ionization (ESI) ion source. High-resolution mass spectra were obtained on a Thermo Fisher Q-Exactive Orbitrap system in positive mode with an ESI ion source.

HPLC Methods

Method A (Analytical HPLC)

Analytical reversed-phase high-performance liquid chromatography (HPLC) was carried out using a Thermo Scientific UltiMate 3000 HPLC system with a Phenomenex Luna C18(2) reversed-phase column (5 μm, 250 mm × 4.6 mm). The UV absorbance was recorded at 214 and 250 nm. Radioactivity was recorded using a Model 105 radio-detector (Carroll & Ramsey Associates, Berkeley, CA). The mobile phases were A, H_2O with 0.1% TFA and B, MeCN with 0.1% TFA. The gradient was as follows: 0–1 min, 100% A; 1–11 min, 0–100% B; 11–16.5 min, 100% B; 16.5–17.5 min, 100–0% B; and 17.5–20 min, 100% A, at a flow rate of 1 mL/min.

Method B (Semi-preparative HPLC)

Purification of the fluorination precursor **11** was conducted on a Thermo Scientific UltiMate 3000 HPLC system with a Phenomenex Luna C18(2) reversed-phase column (5 μm, 250 mm × 10 mm). The UV absorbance was recorded at 214 and 254 nm. The mobile phases were A, H_2O and B, MeCN. The gradient was as follows: 0–3 min, 100% A; 3–20 min, 0–100% B; 20–27.5 min, 100% B; 27.5–30 min, 100–0% B; and 30–33 min, 100% A, at a flow rate of 3 mL/min.

Method C (Semi-preparative HPLC)

Purification of 22-[^{18}F]FDHA was performed on a Knauer HPLC system with an Azura P 6.1L HPG pump and BlueShadow 40D UV/VIS detector with a Phenomenex Luna C18(2) reversed-phase column (5 μm, 250 mm × 10 mm). The UV absorbance was recorded at 254 nm. Radioactivity was recorded using a Model 101 radio-detector (Carroll & Ramsey Associates, Berkeley, CA). The mobile phases were A, H_2O and B, MeCN. The gradient was as follows: 0–3 min, 100% A; 3–20 min, 0–100% B; 20–27.5 min, 100% B; 27.5–30 min, 100–0% B; and 30–33 min, 100% A, at a flow rate of 3 mL/min.

Synthetic Procedures

Methyl 9-Hydroxynona-4,7-diynoate (**1**)

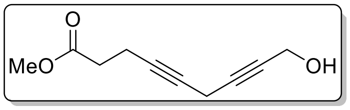

A procedure from the literature[1] was modified as follows: to a solution of methyl 4-pentynoate (617.2 mg, 1.2 equiv) in anhyd. *N*,*N*-dimethylformamide (DMF, 10 mL) was added NaI (900.1 mg, 1.2 equiv), CuI (1.14 g, 1.2 equiv), and Cs_2CO_3 (2.45 g, 1.5 equiv) with rapid stirring. To the mixture was then added a solution of 4-chlorobutyn-1-ol (523.0 mg,) in anhyd. DMF (2 mL). Stirring was continued overnight, then the mixture was diluted with ethyl acetate (EtOAc, 40 mL). To this was then added a mixture of saturated aq. NH_4Cl (40 mL) and saturated aq. $Na_2S_2O_3$ (10 mL). The biphasic mixture was separated, and the aqueous layer was then extracted with EtOAc (2 × 25 mL) and the combined organic layers were washed with water (20 mL), then saturated aq. NaCl (brine, 10 mL). The organic solution was then dried over anhyd. $MgSO_4$ and evaporated under reduced pressure. The residue was loaded onto silica gel and purified by flash chromatography (12 g silica, 0–50% EtOAc in hexanes) to obtain diyne **1** as a yellow oil (746.6 mg, 83% yield). Spectral data agree with those in the literature[1]:

TLC: R_f = 0.41 (1:1 EtOAc/hexanes). ^{1}H NMR (500 MHz, $CDCl_3$): δ 4.24 (t, *J* = 2.2 Hz, 2H), 3.69 (s, 3H), 3.16 (p, *J* = 2.3 Hz, 2H), 2.57 – 2.42 (m, 4H). ^{13}C NMR (125 MHz, $CDCl_3$): δ 172.61, 80.48, 79.09, 78.71, 74.48, 51.94, 51.31, 33.44, 14.72, 9.95.

Methyl 9-Bromonona-4,7-diynoate (**2**)

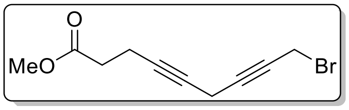

A procedure from the literature[1] was modified as follows: to a solution of **1** (519.4 mg, 2.88 mmol) and CBr_4 (1.44 mg, 1.5 equiv) in anhyd. dichloromethane (DCM, 7.5 mL), cooled in an ice-water bath, was added a solution of triphenylphosphine (PPh_3, 1.14 mg, 1.5 equiv) in anhyd. DCM (2.5 mL) with stirring. After 1.5 h, the solution was evaporated under reduced pressure then the residue was loaded onto silica gel. After flash chromatography (12 g silica, 0–20% EtOAc in hexanes),

bromide **2** was obtained as a yellow oil (559.4 mg, 80% yield). Spectral data agree with those in the literature[1]:

TLC: R_f = 0.73 (1:1 EtOAc/hexanes). ^{1}H NMR (400 MHz, $CDCl_3$): δ 3.90 (t, *J* = 2.3 Hz, 2H), 3.70 (s, 3H), 3.20 (p, *J* = 2.4 Hz, 2H), 2.59 – 2.41 (m, 4H). ^{13}C NMR (100 MHz, $CDCl_3$): δ 172.56, 81.78, 79.36, 75.55, 73.90, 51.97, 33.39, 14.92, 14.73, 10.23.

Methyl 12-Hydroxydodeca-4,7,10-triynoate (**3**)

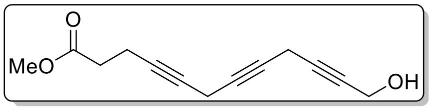

Following a similar procedure for the synthesis of **1**, the reaction of compound **2** (559.4 mg, 2.30 mmol), propargyl alcohol (195 mg, 2 equiv), CuI (658 mg, 1.2 equiv), NaI (520 mg, 1.2 equiv), and Cs_2CO_3 (1.50 g, 1.5 equiv) in DMF (8 mL), followed by aqueous work-up and flash chromatography (12 g silica, 0–50% EtOAc in hexanes) afforded triyne **3** as a yellow oil (327.7 mg, 65% yield). Spectral data agree with those in the literature[1]:

TLC R_f = 0.42 (1:1 EtOAc/hexanes). ^{1}H NMR (500 MHz, $CDCl_3$): δ 4.25 (t, *J* = 2.1 Hz, 2H), 3.69 (s, 3H), 3.19 (p, *J* = 2.3 Hz, 2H), 3.11 (p, *J* = 2.3 Hz, 2H), 2.55 – 2.44 (m, 4H). ^{13}C NMR (125 MHz, $CDCl_3$): δ 172.63, 80.09, 78.93, 78.89, 75.27, 74.69, 74.02, 51.94, 51.32, 33.48, 14.75, 9.99, 9.86. MS (ESI) *m/z*: $[M+H]^+$ Calcd for $C_{13}H_{15}O_3$ 219.1; Found. 219.2.

Methyl 12-Bromododeca-4,7,10-triynoate (**4**)

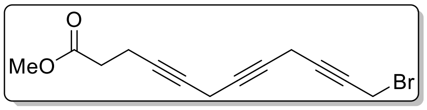

Following a similar procedure for the synthesis of **2**, the reaction of compound **3** (327.7 mg, 1.5 mmol), CBr_4 (745.1 mg, 1.5 equiv), and triphenylphosphine (707.3 mg, 1.5 equiv) in DCM (11 mL), followed by flash chromatography (4 g silica, 0–20% EtOAc in hexanes) afforded the bromide **4** as a yellow oil (259.1 mg, 61% yield). Spectral data agree with those in the literature[1]:

TLC: R_f = 0.75 (1:1 hexanes/EtOAc). ^{1}H NMR (400 MHz, $CDCl_3$): δ 3.90 (t, *J* = 2.3 Hz, 2H), 3.70 (s, 3H), 3.22 (p, *J* = 2.3 Hz, 2H), 3.12 (p, *J* = 2.3 Hz, 2H), 2.57 – 2.42 (m, 4H). ^{13}C NMR (100

MHz, $CDCl_3$): δ 172.57, 81.37, 78.99, 75.75, 75.51, 74.59, 73.51, 51.95, 33.47, 14.83, 14.76, 10.27, 9.89

6-(Trimethylsilyl)hexa-2,5-diyn-1-ol (**5**)

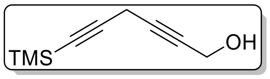

Following a similar procedure for the synthesis of **1**, 3-bromo-1-(trimethylsilyl)-1-propyne (1.05 g, 3 mmol), propargyl alcohol (257.6 mg, 1.5 equiv), CuI (693.4 g, 1.2 equiv), NaI (546 mg, 1.2 equiv), and Cs_2CO_3 (1.47 g, 1.5 equiv) in DMF (9 mL), followed by aqueous work-up and flash chromatography (12 g silica, 0–40% EtOAc in hexanes) afforded diyne **5** as a light-yellow oil (468.3 mg, 94% yield). Spectral data agree with those in the literature[1]:

TLC: R_f = 0.63 (1:1 EtOAc/hexanes), R_f = 0.29 (1:4 EtOAc/hexanes). ^{1}H NMR (400 MHz, $CDCl_3$): δ 4.26 (t, *J* = 2.2 Hz, 2H), 3.25 (t, *J* = 2.2 Hz, 2H), 0.15 (s, 9H). ^{13}C NMR (100 MHz, $CDCl_3$): δ 99.47, 85.61, 79.81, 78.94, 51.36, 11.08, -0.01.

6-(Trimethylsilyl)hexa-2,5-diyn-1-yl 4-methylbenzenesulfonate (**6**)

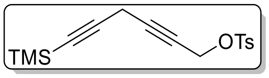

To a solution of **5** (410.0 mg, 2.47 mmol) in anhyd. DCM (8 mL), cooled in an ice-water bath, was added pyridine (280 µL, 1.4 equiv) and *p*-toluenesulfonic anhydride (freshly recrystallized from hot EtOAc, 1.04 g, 1.3 equiv). The mixture was slowly warmed to room temperature. After 1 h, the mixture was partitioned between DCM (15 mL) and saturated aq. NH_4Cl (15 mL). The aqueous layer was extracted with DCM (2 × 10 mL) and the pooled organic solutions were washed with brine. The organic solution was dried over anhyd. Na_2SO_4, then evaporated under reduced pressure. The residue was loaded onto silica gel and after flash chromatography (12 g silica, 0–20% EtOAc in hexanes), tosylate **6** was obtained as a yellow oil (471.7 mg, 60% yield). Spectral data agree with those in the literature[1]:

TLC: R_f = 0.43 (1:4 EtOAc/hexanes), R_f = 0.74 (1:1 EtOAc/hexanes). ^{1}H NMR (400 MHz, $CDCl_3$): δ 7.82 (d, *J* = 8.4 Hz, 2H), 7.35 (d, *J* = 7.9 Hz, 2H), 4.70 (t, *J* = 2.2 Hz, 2H), 3.11 (t, *J* = 2.2 Hz, 3H), 2.46 (s, 3H), 0.16 (s, 9H). ^{13}C NMR (100 MHz, $CDCl_3$): δ 145.15, 133.33, 129.94, 128.31, 98.27, 86.06, 83.60, 72.61, 58.32, 21.82, 11.08, -0.03.

10-(Trimethylsilyl)deca-3,6,9-triyn-1-ol (**7**)

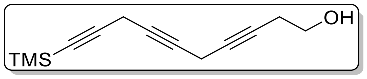

Following a similar procedure for the synthesis of **1**, the reaction of compound **6** (1.25 g, 3.9 mmol), 3-butyn-1-ol (361.6 mg, 1.5 equiv), CuI (898 mg, 1.2 equiv), NaI (704 mg, 1.2 equiv), and Cs_2CO_3 (1.91 g, 1.5 equiv) in DMF (12 mL), followed by aqueous work-up and flash chromatography (12 g silica, 0–30% EtOAc in hexanes) afforded triyne **7** as a yellow oil (409.9 mg, 48% yield). Spectral data agree with those in the literature[1]:

TLC: R_f = 0.57 (1:1 EtOAc/hexanes), R_f = 0.19 (1:4 EtOAc/hexanes). ^{1}H NMR (400 MHz, $CDCl_3$): δ 3.70 (t, *J* = 6.2 Hz, 2H), 3.20 (t, *J* = 2.4 Hz, 2H), 3.16 (p, *J* = 2.4 Hz, 2H), 2.44 (tt, *J* = 6.2, 2.4 Hz, 2H), 1.77 (s, 1H), 0.16 (s, 9H). ^{13}C NMR (100 MHz, $CDCl_3$): δ 99.90, 85.35, 77.39, 76.24, 75.06, 74.19, 61.23, 23.24, 11.05, 10.00, 0.04.

Deca-3,6,9-triyn-1-ol (**8**)

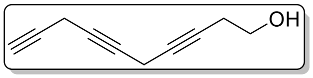

A procedure from the literature[1] was modified as follows: compound **7** (409.9 mg, 1.88 mmol) was dissolved in anhyd. tetrahydrofuran (THF, 10 mL) and cooled in an ice-water bath. Acetic acid (65 µL, 0.6 equiv) was added to a 1 M solution of tetrabutylammonium fluoride (TBAF) in THF (2.25 mL, 1.2 equiv) and this mixture was then added dropwise with stirring over 5 min to the cooled THF solution of **7**. The resulting brown solution was stirred for 1 h, and then partitioned between diethyl ether (40 mL) and cold water (40 mL). The aqueous layer was further extracted with diethyl ether (3 × 15 mL) and the pooled organic layers were washed with water (20 mL) and brine (20 mL). The organic solution was dried over anhyd. Na_2SO_4 and evaporated under reduced pressure. The residue was loaded onto silica gel and after flash chromatography (4 g silica, 0–40% EtOAc in hexanes), desilylated triyne **8** was obtained as a light-yellow oil (229.5 mg, 84% yield). Spectral data agree with those in the literature[1]:

TLC: R_f = 0.51 (1:1 EtOAc/hexanes), R_f = 0.14 (1:4 EtOAc/hexanes). ^{1}H NMR (400 MHz, $CDCl_3$): δ 3.69 (t, *J* = 6.3 Hz, 2H), 3.18 – 3.11 (m, 4H), 2.43 (tt, *J* = 6.3, 2.3 Hz, 2H), 2.06 (t, *J* = 2.6 Hz,

1H), 1.99 (s, 1H). ^{13}C NMR (100 MHz, $CDCl_3$): δ 78.20, 77.48, 76.01, 75.33, 73.84, 68.97, 61.17, 23.17, 9.88, 9.69.

Methyl (4Z,7Z,10Z,13Z,16Z,19Z)-22-(Tosyloxy)docosa-4,7,10,13,16,19-hexaenoate (**11**)

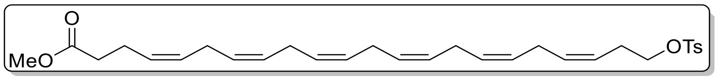

Following a similar procedure for the synthesis of **1**, the reaction of **4** (259.1 mg, 0.92 mmol), **8** (229.5 mg, 1.7 equiv), CuI (211.1 mg, 1.2 equiv), NaI (166.0 mg, 1.2 equiv), and Cs_2CO_3 (452.0 mg, 1.5 equiv) in DMF (6 mL), followed by aqueous work-up and flash chromatography (12 g silica, 0–50% EtOAc in hexanes) afforded hexayne **9** as a yellow-orange solid (130.5 mg, 41% yield). Due to the instability of **9**, it was used in the following step immediately without further characterization by NMR.

A procedure from the literature[1] was modified as follows: to a flask charged with **9** (130.5 mg, 0.38 mmol) was added a mixture of methanol (4 mL), EtOAc (4 mL), and 2-methyl-2-butene (8 mL). The solution was immediately purged with N_2 and to it was then added Lindlar's catalyst (130.2 mg) and pyridine (2 mL). H_2 gas was bubbled through the suspension for 1 min, which was then kept stirring under a constant positive-pressure atmosphere of H_2 (balloon pressure, with a 26G needle through a rubber septum to vent) while monitoring the progress of the hydrogenation by TLC (1:1 EtOAc/hexanes). After 2 h, the mixture was filtered, and the filtrate was then partitioned between EtOAc (30 mL) and 1 M aq. HCl (25 mL). The aqueous layer was further extracted with EtOAc (3 ×15 mL), and the pooled organic solutions were washed with water (20 mL) and then brine (15 mL). The organic phase was then dried over anhyd. Na_2SO_4 and evaporated under reduced pressure yielding a brown oil. The residue was loaded onto silica gel and after flash chromatography (4 g silica, 0–25% EtOAc in hexanes), the hydrogenated product was obtained as a light-yellow oil (79.2 mg, 59% crude yield). Analytical HPLC of the isolated products showed that it was a mixture of the intended hexaene product **10** (*ca.* 70% by UV at 250 nm) with over-reduced and under-reduced derivatives; the mixture was used in the next step without further purification. In smaller-scale reactions, the crude product may be isolated from strongly colored impurities by taking the residue up in 1:4 EtOAc/hexanes, passing the solution through a short silica plug, and eluting with 1:4 EtOAc/hexanes.

A procedure was adapted from the literature[2] as follows: to a solution of the partially purified **10** (17.5 mg) in anhyd. DCM (3 mL), cooled in an ice-water bath, was added triethylamine (14 µL) and 4-(dimethylamino)pyridine (0.8 mg). Then, a solution of tosyl chloride (34.7 mg) in DCM (0.5 mL) was added to the mixture. After slowly warming to room temperature and then continuing stirring for 2 days, the solution was diluted with DCM (10 mL). The solution was then washed with cold water (10 mL), then with brine (5 mL). The organic solution was then dried over anhyd. Na_2SO_4 and evaporated under reduced pressure. The residue was loaded onto silica gel and partially purified by flash chromatography (4 g silica, 0–10% EtOAc in hexanes) to obtain the desired tosylated compound. After further purification of the isolated product by semi-preparative HPLC (Method B), analytically pure hexaene tosylate **11** was obtained as a colorless oil (5.6 mg, 13% yield over 2 steps):

TLC: R_f = 0.37 (1:4 EtOAc/hexanes), R_f = 0.14 (1:9 EtOAc/hexanes), R_f = 0.03 (1:19 EtOAc/hexanes). Analytical HPLC (Method A): t_r = 15.72 min. ^{1}H NMR (400 MHz, $CDCl_3$): δ 7.79 (d, J = 8.2 Hz, 2H), 7.34 (d, J = 8.0 Hz, 2H), 5.53 – 5.21 (m, 12H), 4.02 (t, J = 6.9 Hz, 2H), 3.67 (s, 3H), 2.87 – 2.72 (m, 10H), 2.45 (s, 3H), 2.44 – 2.34 (m, 6H). ^{13}C NMR (100 MHz, $CDCl_3$): δ 173.69, 144.85, 133.33, 131.77, 129.96, 129.95, 129.45, 128.66, 128.44, 128.36, 128.28, 128.26, 128.14, 128.06, 128.05, 127.72, 123.50, 69.72, 51.72, 34.15, 27.26, 25.82, 25.78, 25.78, 25.76, 25.72, 22.94, 21.79. HRMS (ESI/QTOF) *m/z*: $[M+H]^+$ Calcd for $C_{30}H_{41}O_5S$ 513.2669; Found 513.2651.

*Methyl 22-Hydroxydocosa-4,7,10,13,16,19-hexaynoate (**9**)*

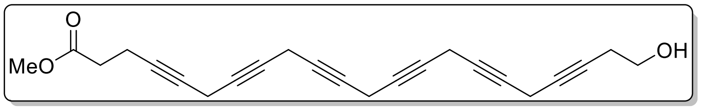

TLC: R_f = 0.42 (1:1 EtOAc/hexanes). Analytical HPLC (Method A): t_r = 12.31 min. MS (ESI) *m/z*: $[M+H]^+$ Calcd for $C_{23}H_{23}O_3$ 347.2; Found 347.2.

*Methyl (4Z,7Z,10Z,13Z,16Z,19Z)-22-Hydroxydocosa-4,7,10,13,16,19-hexaenoate (**10**)*

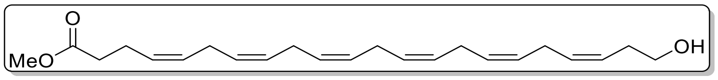

TLC: R_f = 0.69 (1:1 EtOAc/hexanes), R_f = 0.19 (1:4 EtOAc/hexanes). Analytical HPLC (Method A): t_r = 14.79 min. MS (ESI) *m/z*: $[M+H]^+$ Calcd for $C_{23}H_{23}O_3$ 359.3; Found 359.3.

Methyl (4Z,7Z,10Z,13Z,16Z,19Z)-22-Fluorodocosa-4,7,10,13,16,19-hexaenoate (**13**)

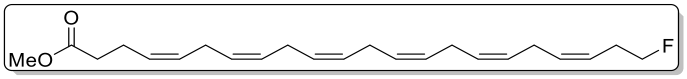

A procedure from the literature[2] was modified as follows: to a solution of **11** (10.7 mg) in anhyd. THF (1.5 mL), cooled in an ice-water bath, was added 1 M TBAF in THF (87 µL, 4.2 equiv). The resulting orange solution was slowly warmed to room temperature with stirring overnight. The solution was evaporated under reduced pressure and the residue was taken up in DCM (5 mL). The solution was washed with water (1 mL) and then brine (1 mL). After drying the solution over anhyd. Na_2SO_4, the solvent was evaporated under reduced pressure and the residue was loaded onto silica gel. After flash chromatography (4 g silica, 0–5% EtOAc in hexanes), two products were isolated as colorless oils: an elimination product **12** (2.2 mg, 29% yield) and the intended substitution product **13** (2.1 mg, 29% yield):

TLC: R_f = 0.39 (1:9 EtOAc/hexanes), R_f = 0.22 (1:19 EtOAc/hexanes). Analytical HPLC (Method A): t_r = 15.49 min. ^1H NMR (400 MHz, $CDCl_3$): δ 5.58 – 5.29 (m, 12H), 4.43 (dt, $^2J_{FH}$ = 47.1 Hz, $^3J_{HH}$ = 6.5 Hz, 2H), 3.67 (s, 3H), 2.92 – 2.72 (m, 10H), 2.49 (dq, $^3J_{FH}$ = 22.3 Hz, $^3J_{HH}$ = 6.8 Hz, 2H), 2.42 – 2.33 (m, 4H). ^{19}F NMR (376 MHz, $CDCl_3$): δ –216.81 (tt, $^2J_{HF}$ = 47.0 Hz, $^3J_{HF}$ = 23.4 Hz).

*Methyl (4Z,7Z,10Z,13Z,16Z,19Z)-Docosa-4,7,10,13,16,19,21-heptaenoate (**12**)*

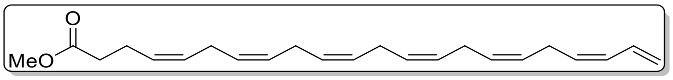

TLC: R_f = 0.46 (1:9 EtOAc/hexanes), R_f = 0.28 (1:19 EtOAc/hexanes). Analytical HPLC (Method A): t_r = 16.29 min. ^1H NMR (400 MHz, $CDCl_3$): δ 6.66 (dt, *J* = 16.9, 10.6 Hz, 1H), 6.02 (t, *J* = 10.9 Hz, 1H), 5.48 – 5.32 (m, 11H), 5.21 (d, *J* = 16.8 Hz, 1H), 5.12 (d, *J* = 10.1 Hz, 1H), 3.67 (s, 3H), 2.97 (t, *J* = 6.8 Hz, 2H), 2.91 – 2.80 (m, 8H), 2.44 – 2.33 (m, 4H).

(4*Z*,7*Z*,10*Z*,13*Z*,16*Z*,19*Z*)-22-Fluorodocosa-4,7,10,13,16,19-hexaenoic acid (22-FDHA)

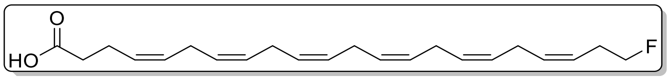

A procedure from the literature[2] was adapted as follows: to a solution of the methyl ester **13** (2.1 mg) in THF (0.85 mL) was added a solution of LiOH (1.1 mg, 8 equiv) in water (0.15 mL). After stirring overnight at room temperature, the mixture was diluted with water (1.0 mL), then the pH was adjusted to around 2 with 0.1 M aq. H_2SO_4. The aqueous mixture was then extracted with DCM (3 × 1 mL) and the pooled organic solutions were washed with brine (1 mL), then dried over anhyd. $MgSO_4$. The solution was evaporated under reduced pressure and the residue was loaded onto silica gel. After flash chromatography (4 g silica, 0–20 % EtOAc in hexanes), 22-FDHA in its free acid form was obtained as a colorless oil (1.9 mg, 94%, yield):

TLC: R_f = 0.13 (1:4 EtOAc/hexanes). Analytical HPLC (Method A): t_r = 14.47 min. ^{1}H NMR (500 MHz, $CDCl_3$): δ 5.63 – 5.25 (m, 12H), 4.43 (dt, $^2J_{FH}$ =47.2 Hz, $^3J_{HH}$ = 6.5 Hz, 2H), 2.92 – 2.71 (m, 10H), 2.49 (dq, $^3J_{FH}$ = 23.0 Hz, $^3J_{HH}$ = 6.8 Hz, 2H), 2.42 – 2.37 (m, 4H). ^{19}F NMR (470 MHz, $CDCl_3$): δ –216.75 (tt, $^2J_{HF}$ = 47.0 Hz, $^3J_{HF}$ = 23.4 Hz). ^{13}C NMR (100 MHz, $CDCl_3$): δ 177.70, 131.25, 129.66, 128.53, 128.41, 128.37, 128.32, 128.30, 128.23, 128.17, 127.99, 127.78, 124.08 (d, $^3J_{FC}$ = 7.3 Hz), 83.32 (d, $^1J_{FC}$ = 167.9 Hz), 33.96, 28.81 (d, $^2J_{FC}$ = 20.7 Hz), 25.86, 25.79, 25.78, 25.77, 25.73, 22.72. HRMS (ESI/QTOF) *m*/*z*: $[M+H]^+$ Calcd for $C_{22}H_{31}FO_2$ 347.2381; Found 347.2371.

NMR Spectra

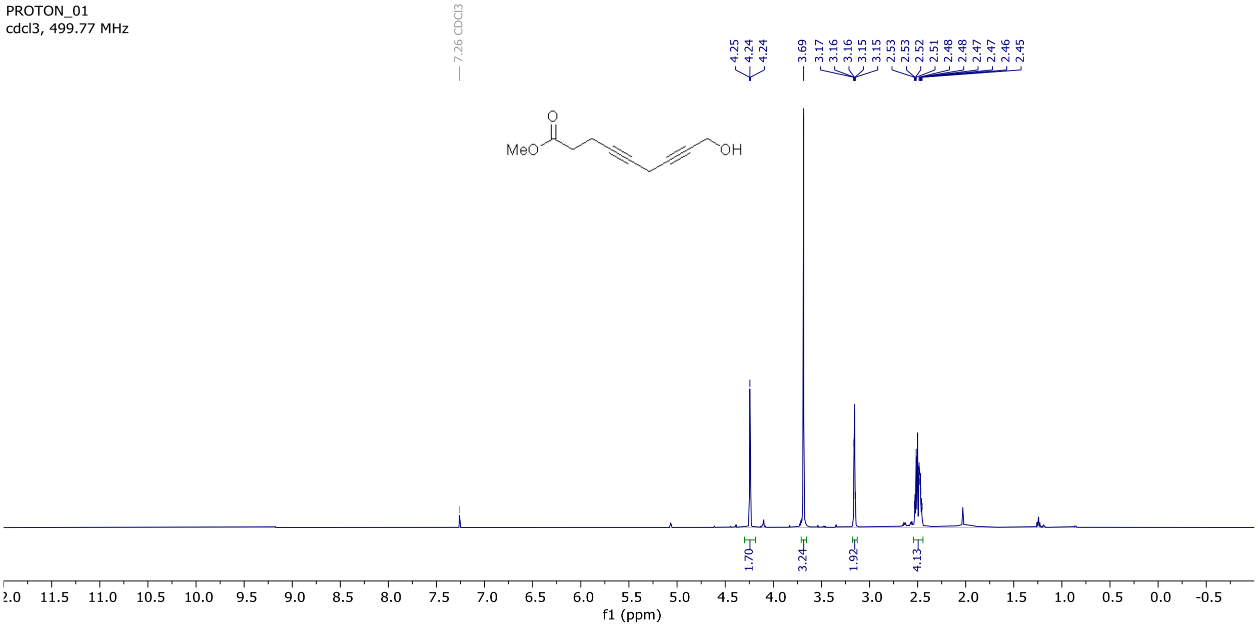

^{1}H NMR ($CDCl_3$, 500 MHz) spectrum of **1**

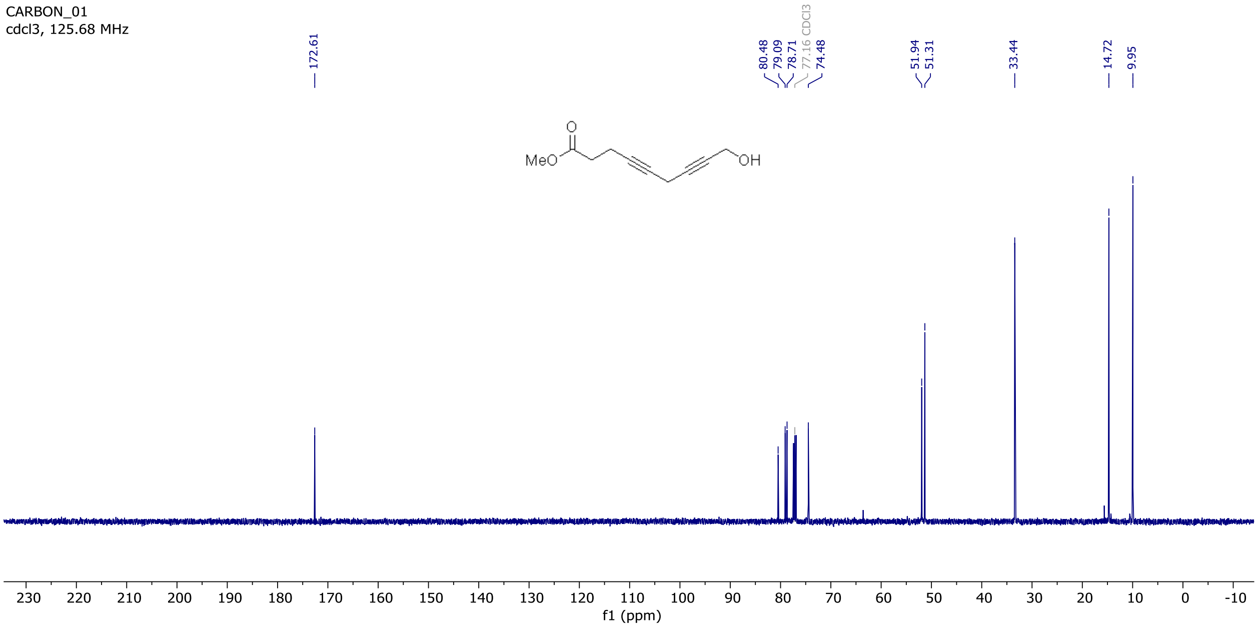

^{13}C{^{1}H} NMR ($CDCl_3$, 125 MHz) spectrum of **1**

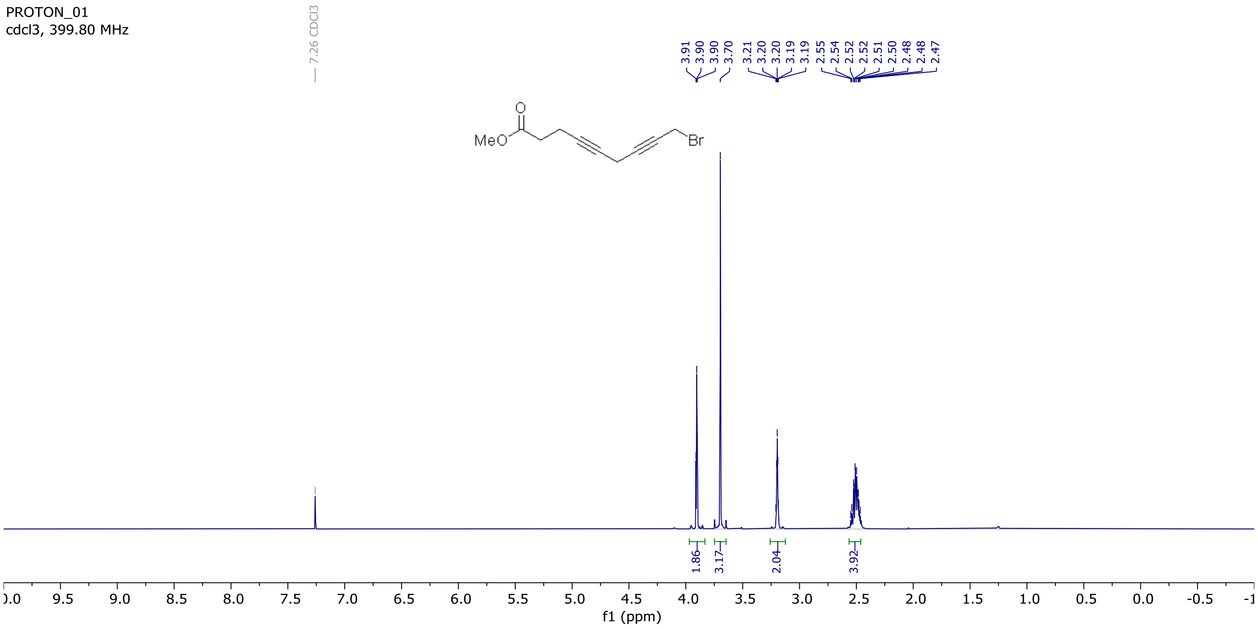

^{1}H NMR ($CDCl_3$, 400 MHz) spectrum of **2**

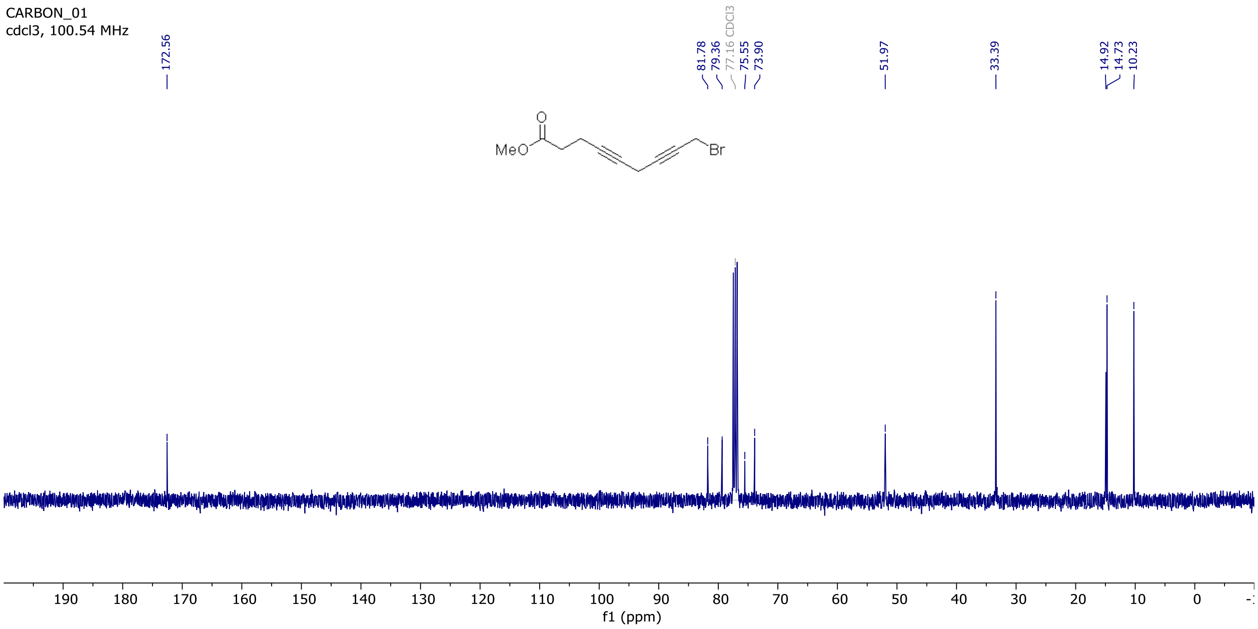

$^{13}C\{^{1}H\}$ NMR ($CDCl_3$, 100 MHz) spectrum of **2**

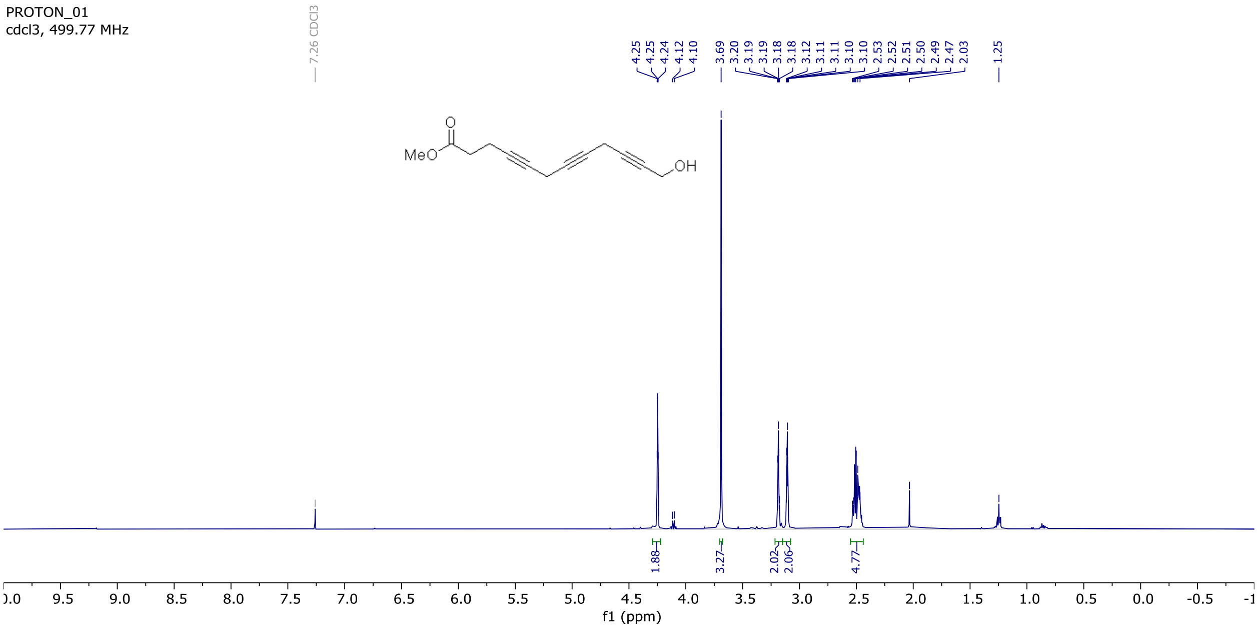

^{1}H NMR ($CDCl_3$, 500 MHz) spectrum of **3**

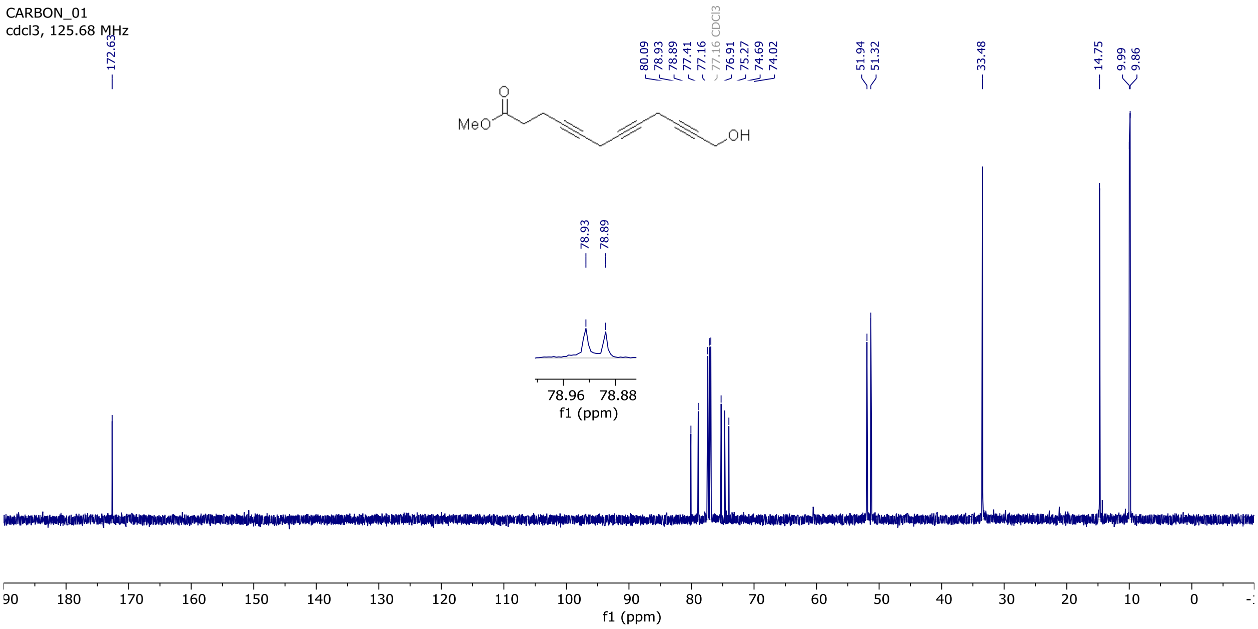

^{13}C{^{1}H} NMR ($CDCl_3$, 125 MHz) spectrum of **3**

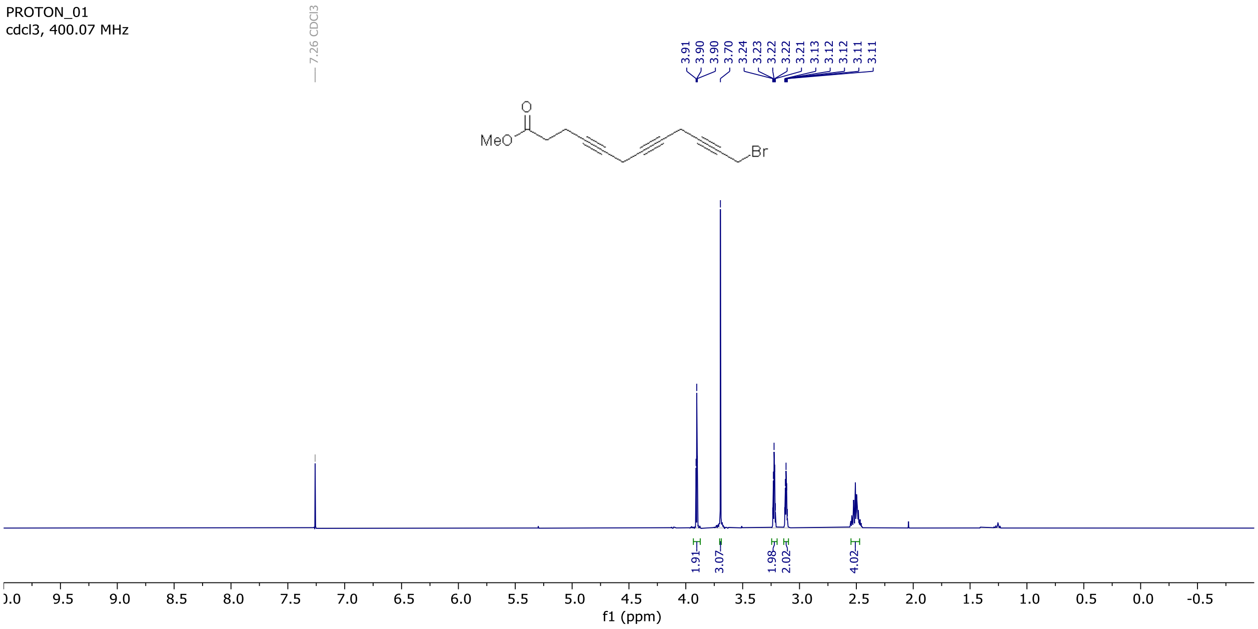

^{1}H NMR ($CDCl_3$, 400 MHz) spectrum of **4**

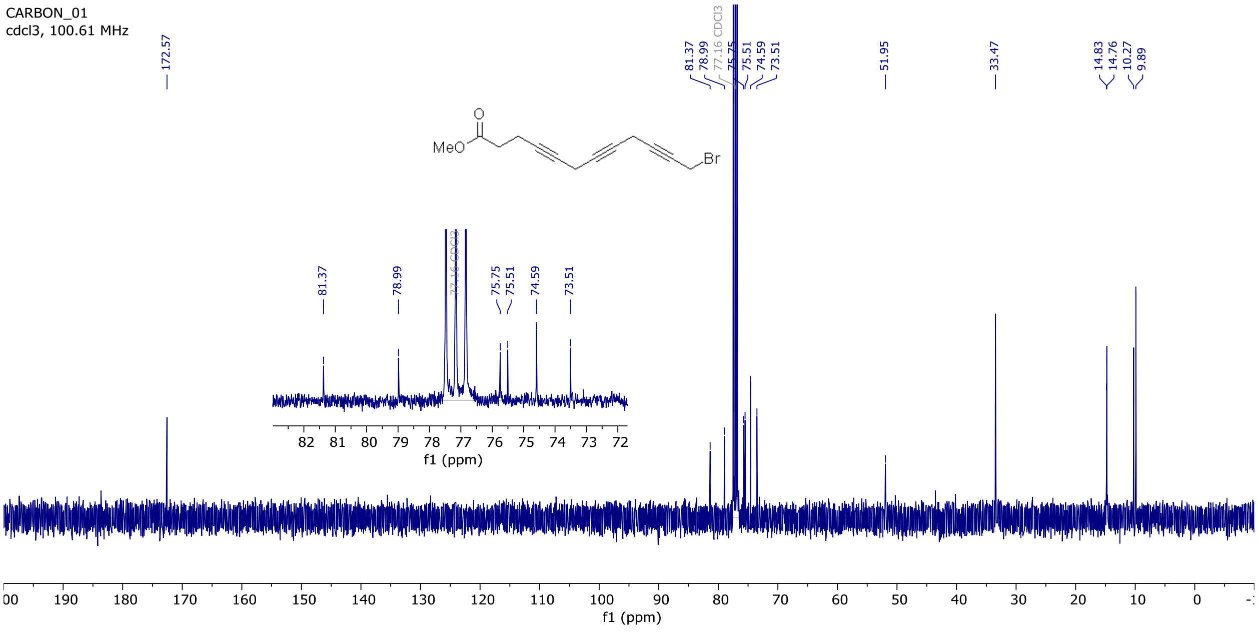

^{13}C{^{1}H} NMR ($CDCl_3$, 100 MHz) spectrum of **4**

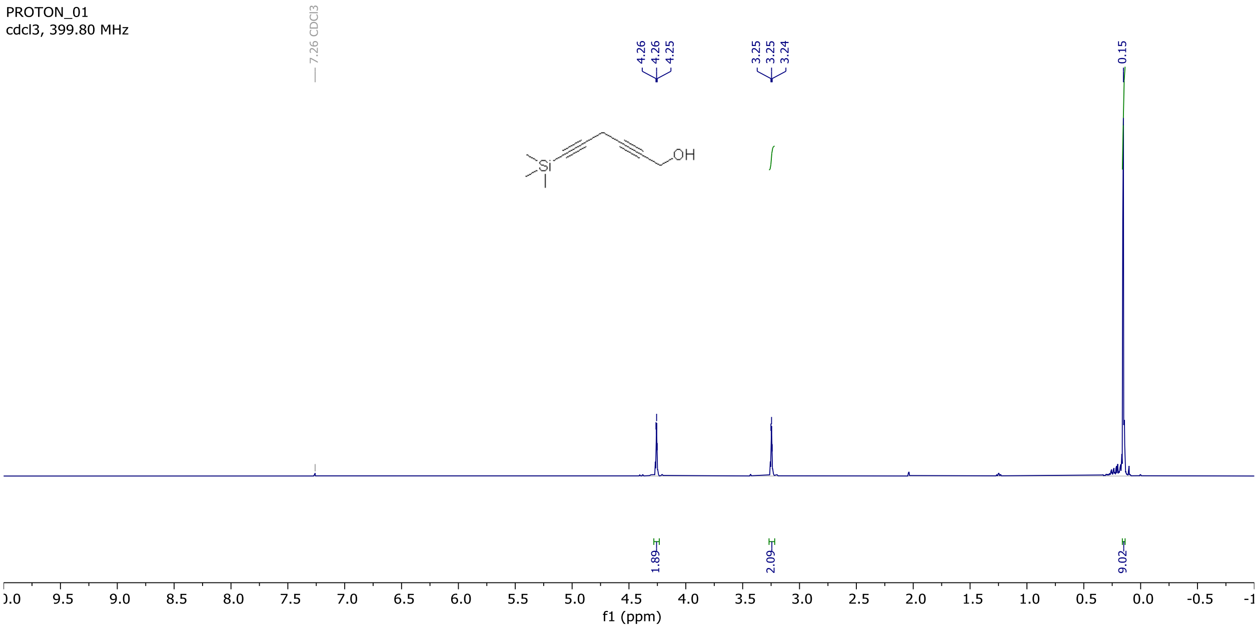

^{1}H NMR ($CDCl_3$, 400 MHz) spectrum of **5**

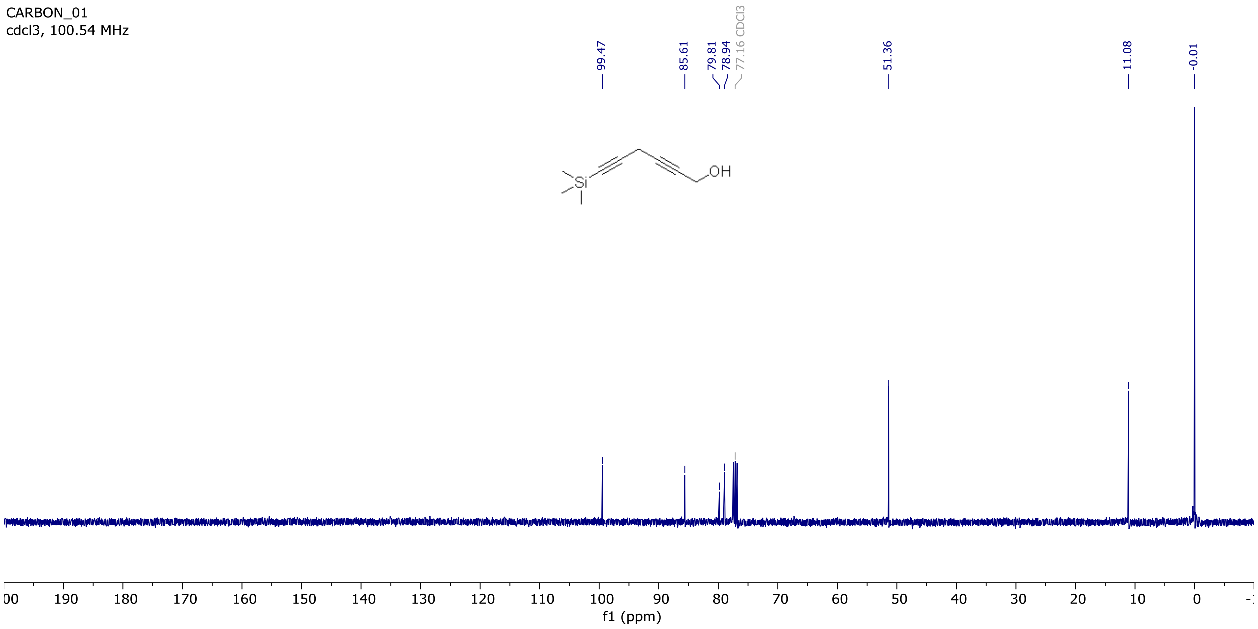

^{13}C{^{1}H} NMR ($CDCl_3$, 100 MHz) spectrum of **5**

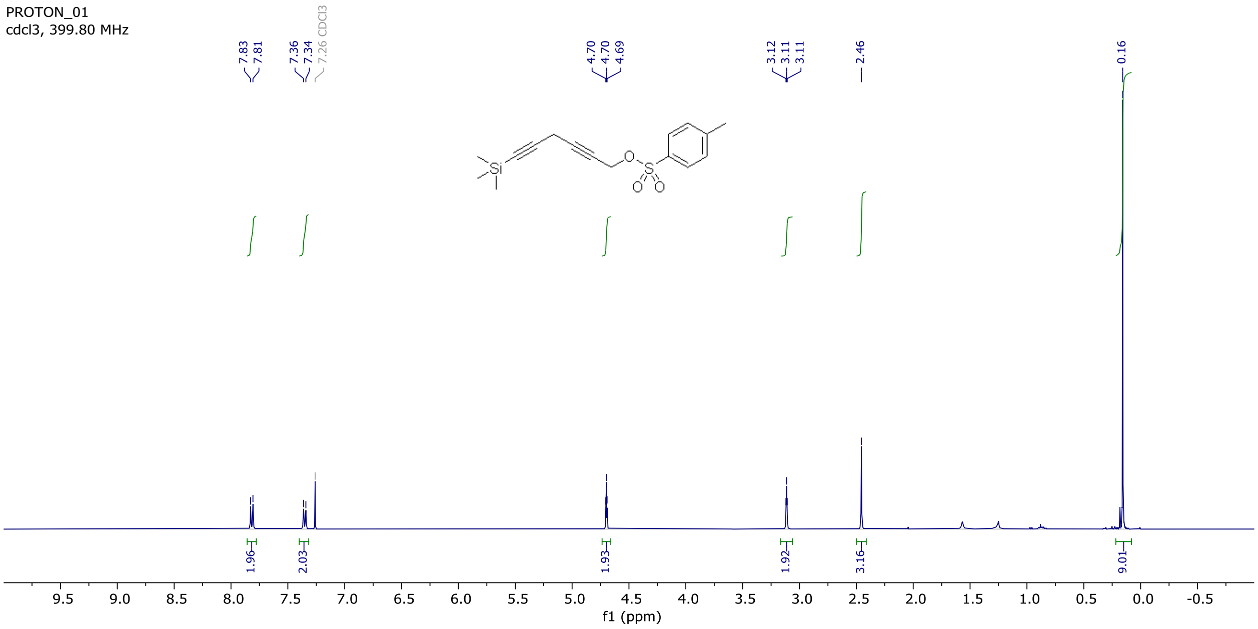

^{1}H NMR ($CDCl_3$, 400 MHz) spectrum of **6**

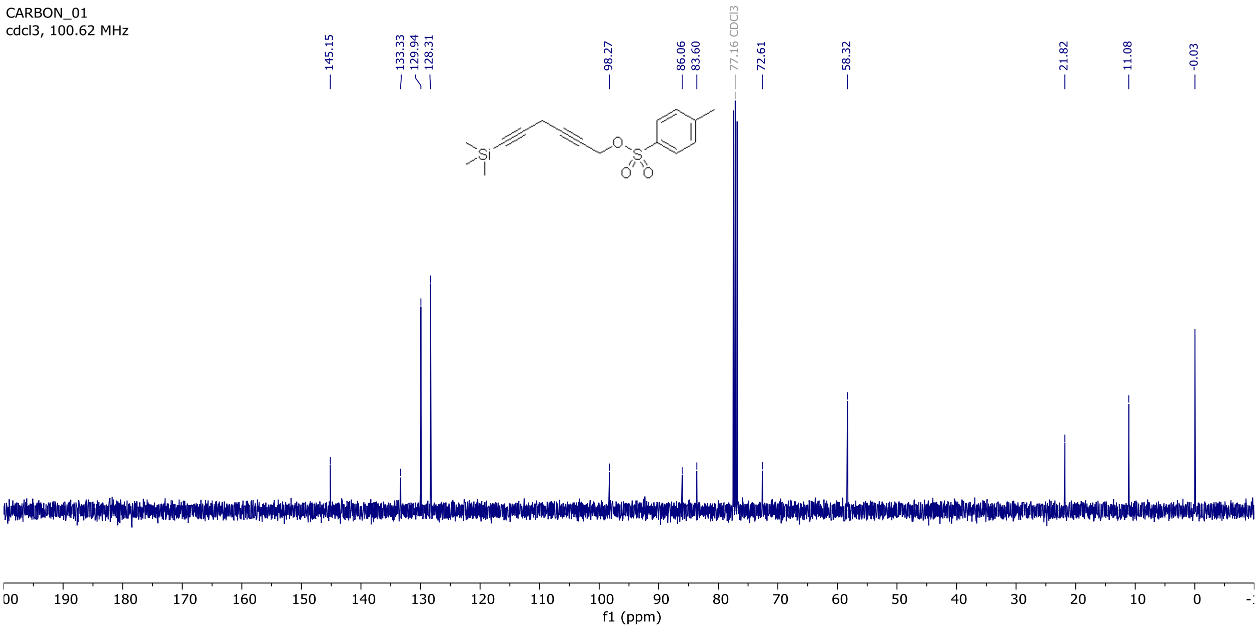

$^{13}C\{^{1}H\}$ NMR ($CDCl_3$, 100 MHz) spectrum of **6**

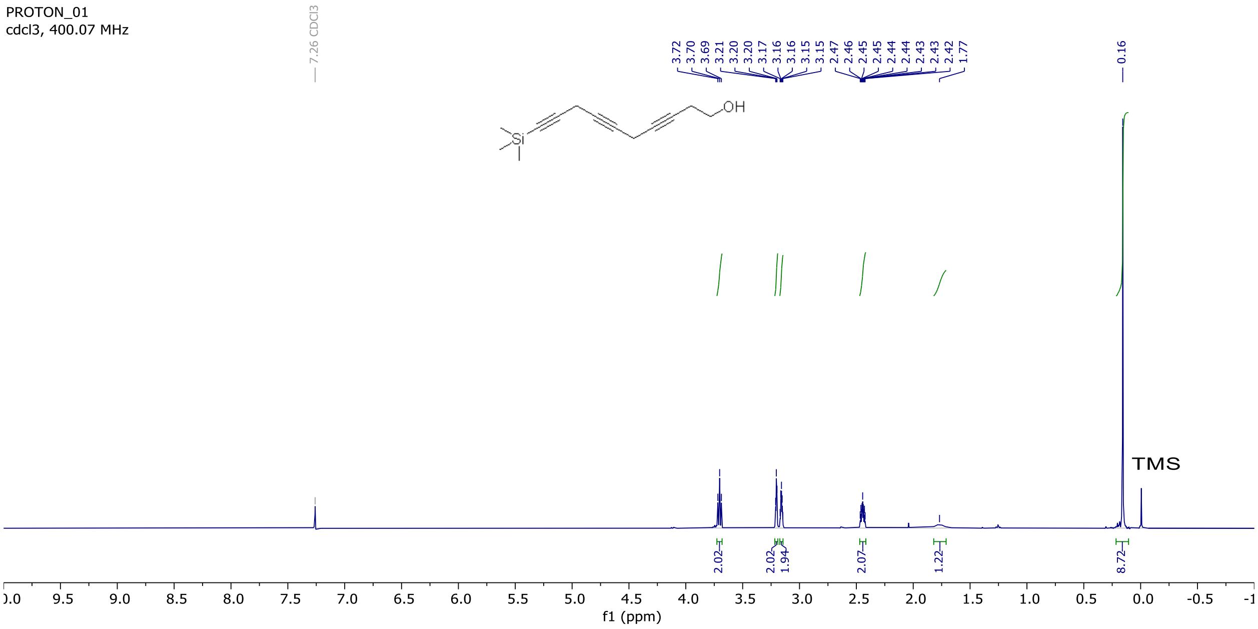

^{1}H NMR ($CDCl_3$, 400 MHz) spectrum of **7**

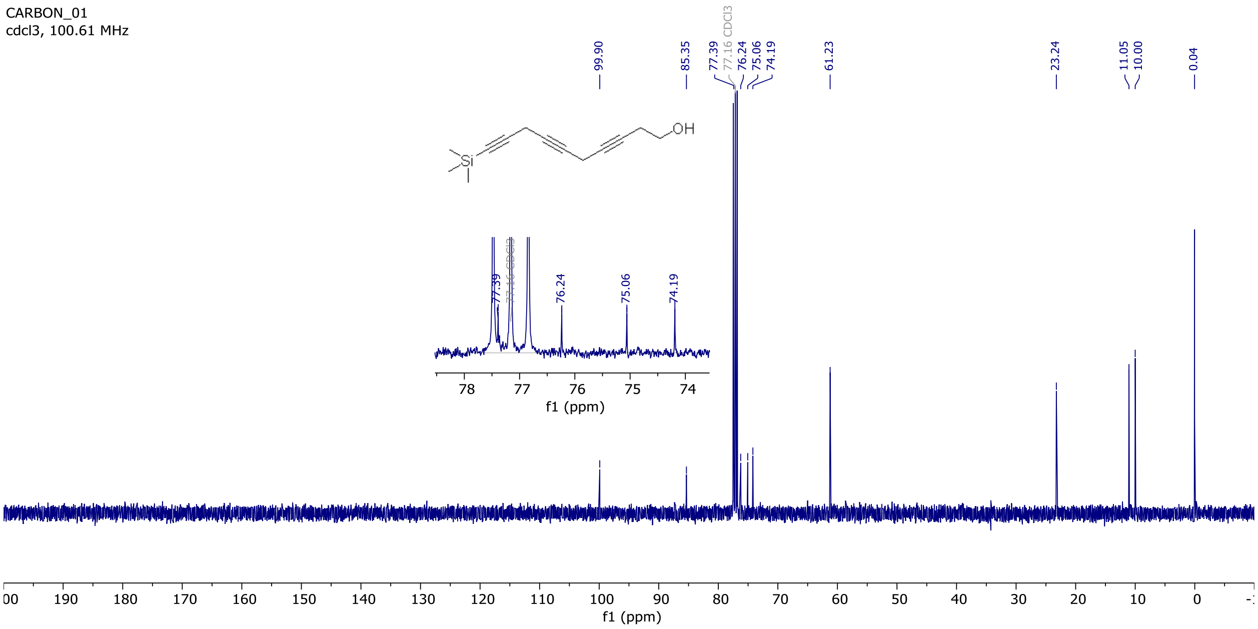

$^{13}C\{^{1}H\}$ NMR ($CDCl_3$, 100 MHz) spectrum of **7**

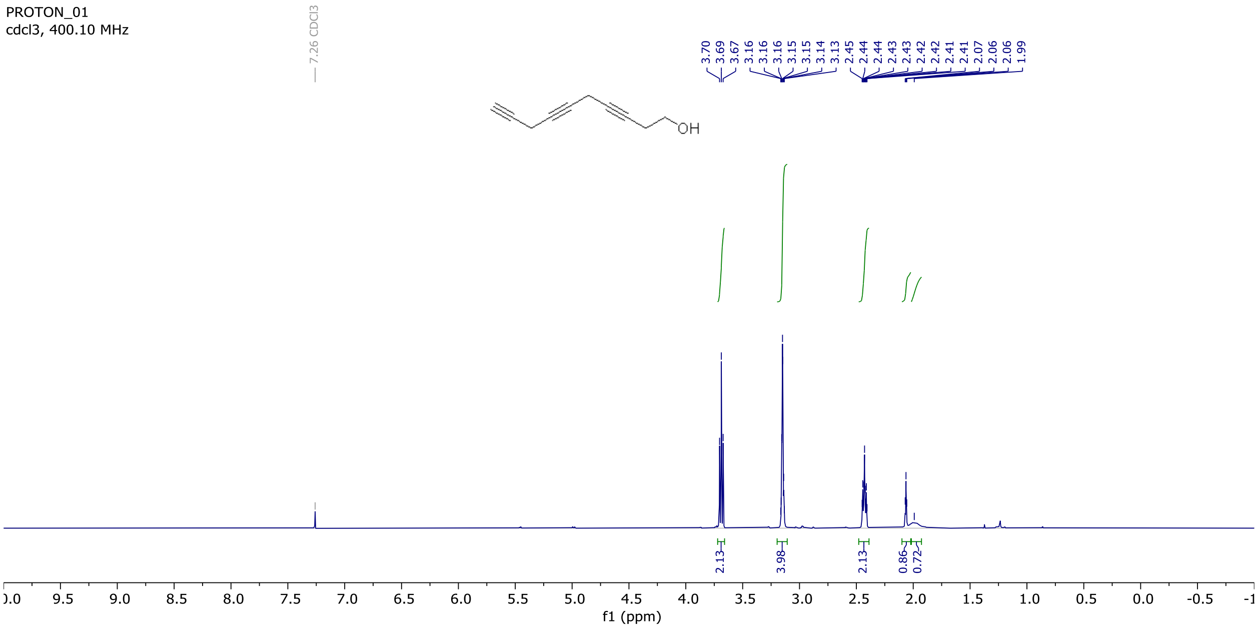

^{1}H NMR ($CDCl_3$, 400 MHz) spectrum of **8**

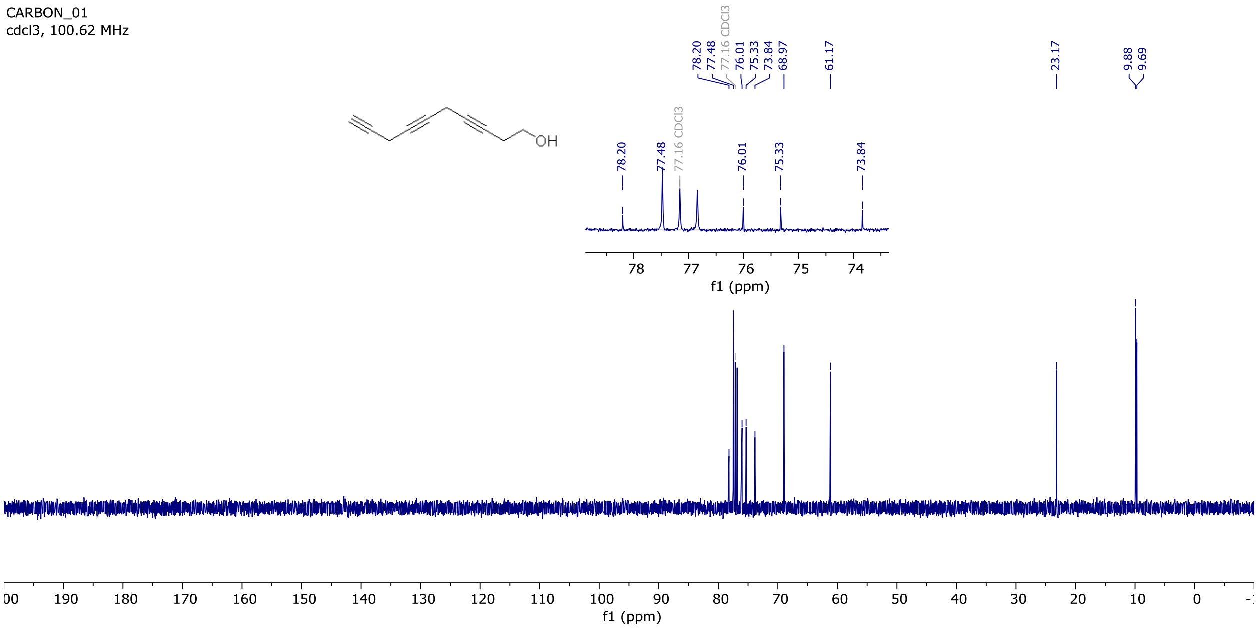

$^{13}C\{^{1}H\}$ NMR ($CDCl_3$, 100 MHz) spectrum of **8**

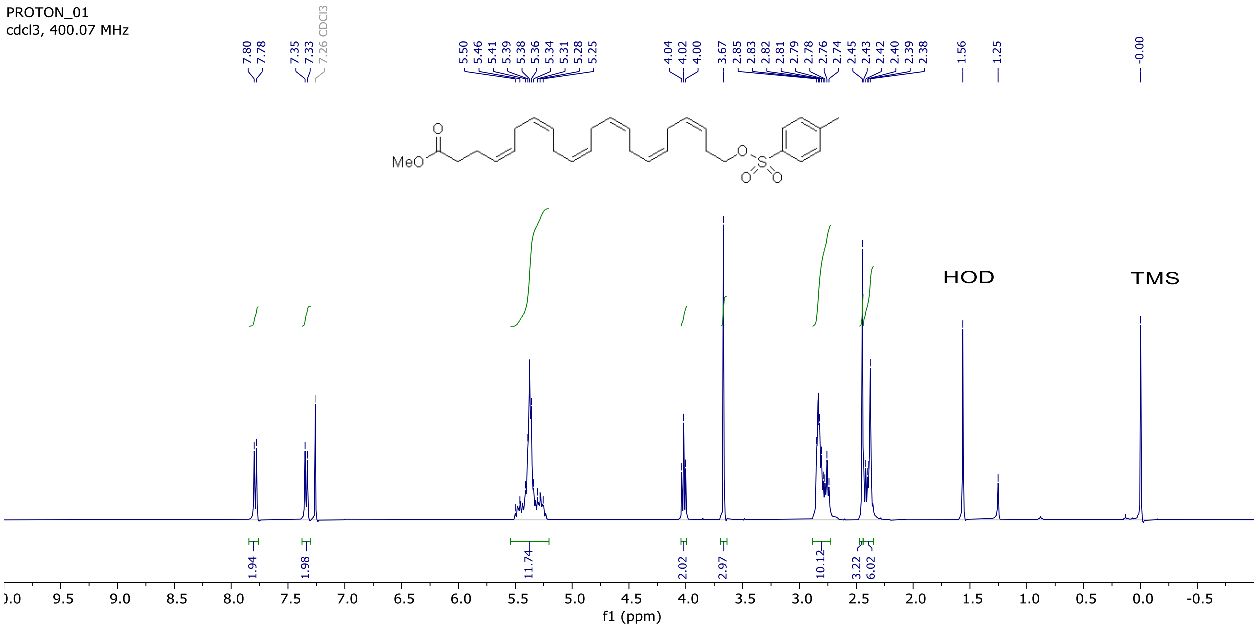

^{1}H NMR ($CDCl_3$, 400 MHz) spectrum of **11**

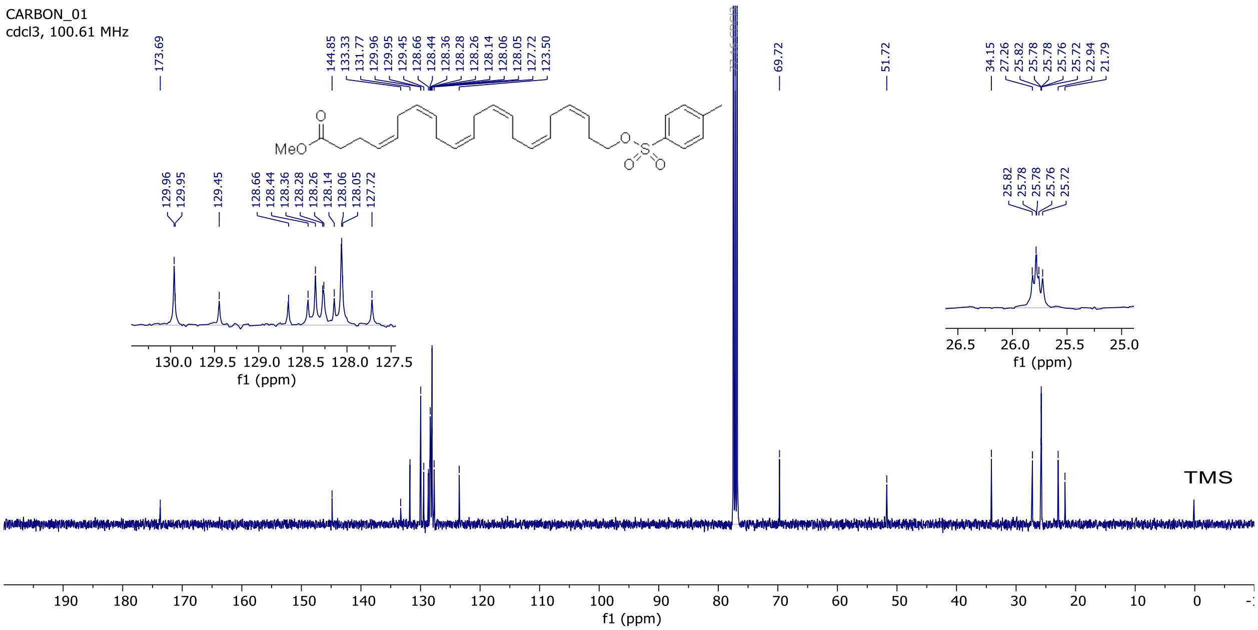

$^{13}C\{^{1}H\}$ NMR ($CDCl_3$, 100 MHz) spectrum of **11**

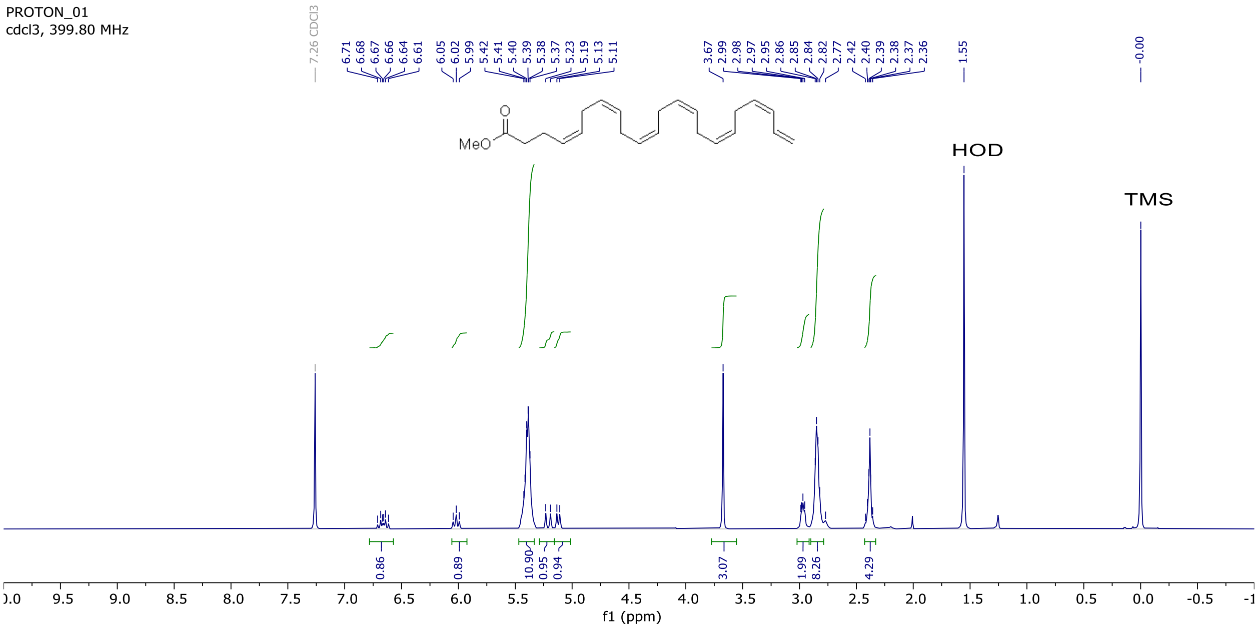

^{1}H NMR ($CDCl_3$, 400 MHz) spectrum of **12**

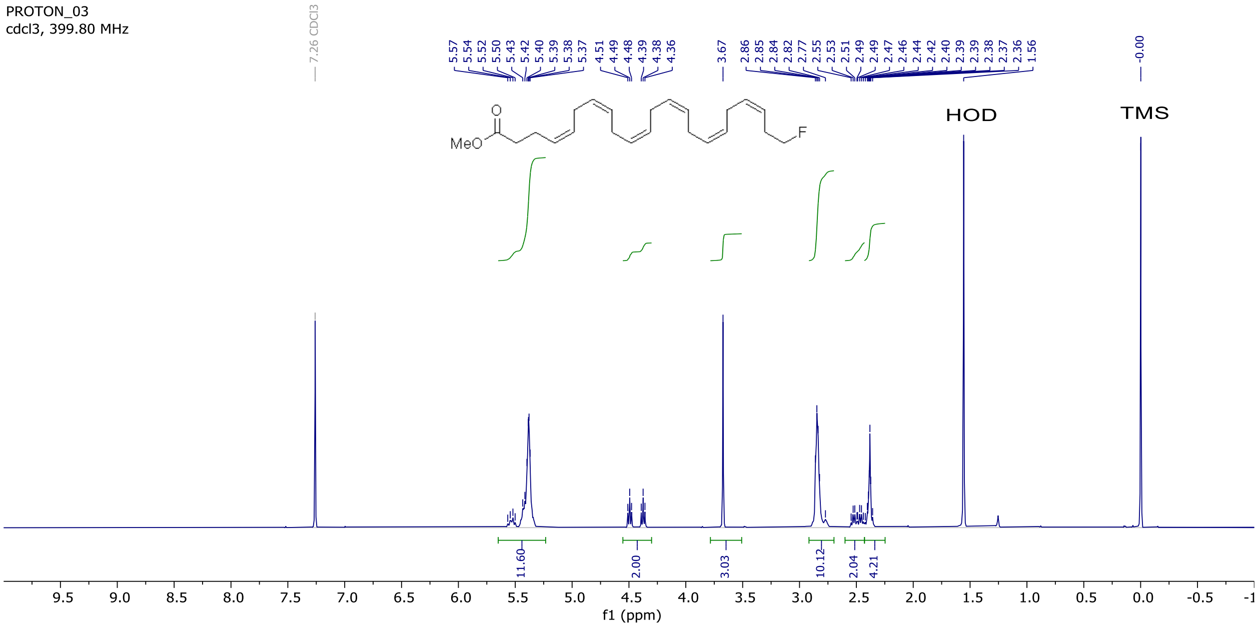

^{1}H NMR ($CDCl_3$, 400 MHz) spectrum of **13**

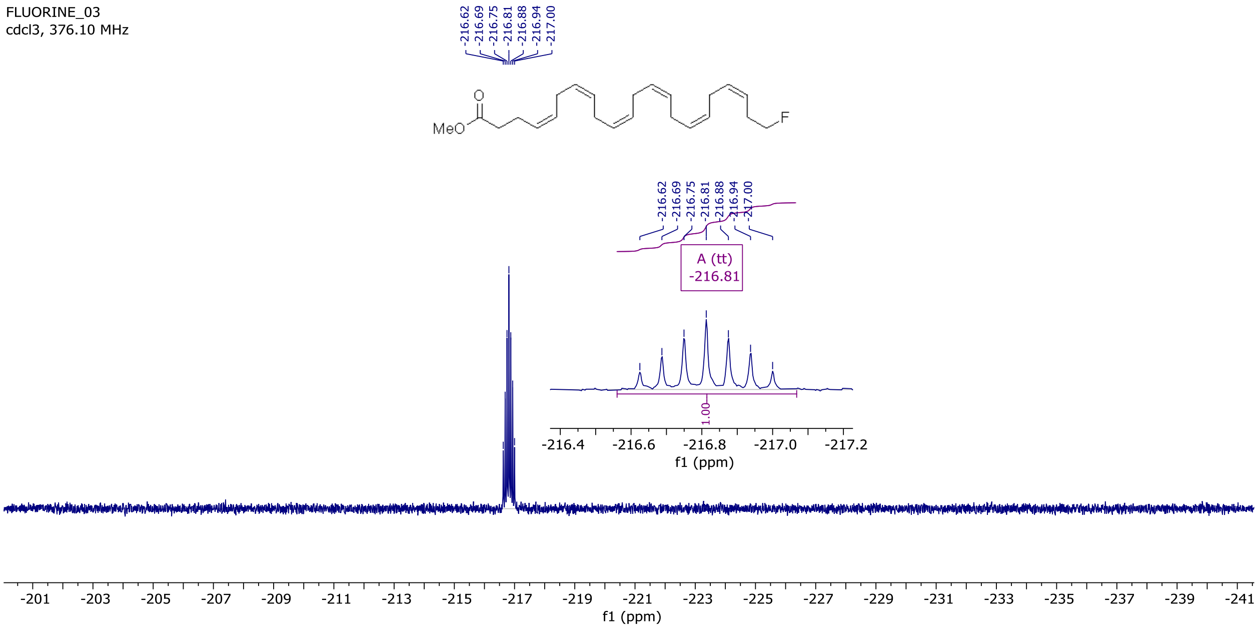

^{19}F NMR ($CDCl_3$, 376 MHz) spectrum of **13**

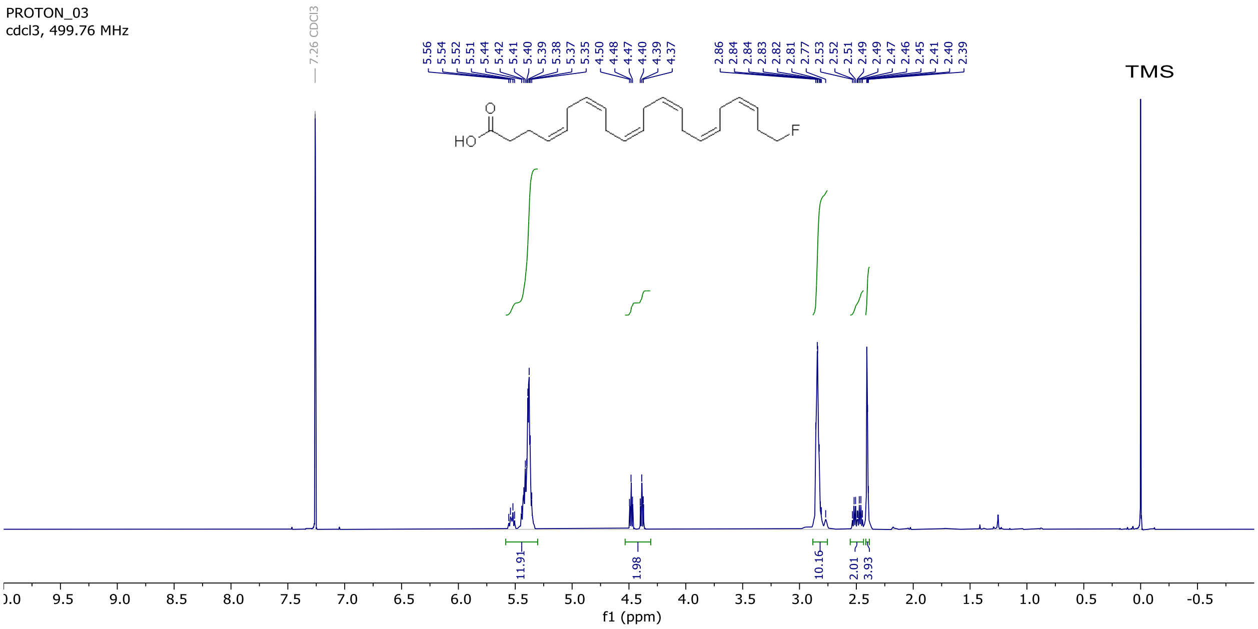

^{1}H NMR ($CDCl_3$, 500 MHz) spectrum of 22-FDHA

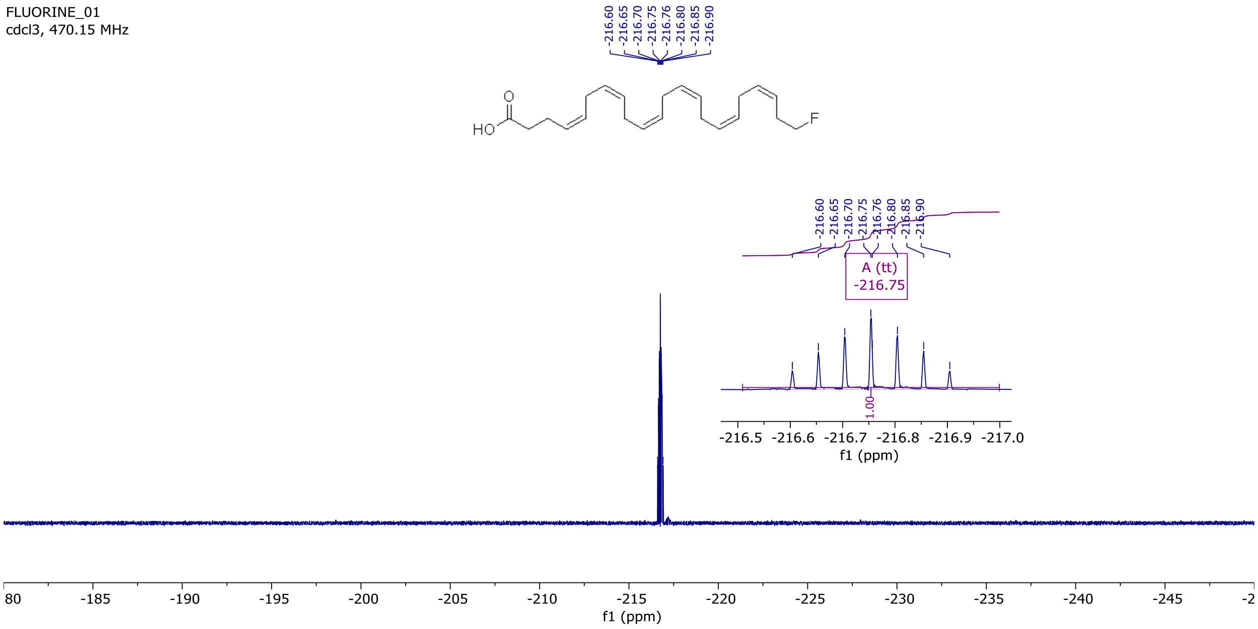

^{19}F NMR ($CDCl_3$, 470 MHz) spectrum of 22-FDHA

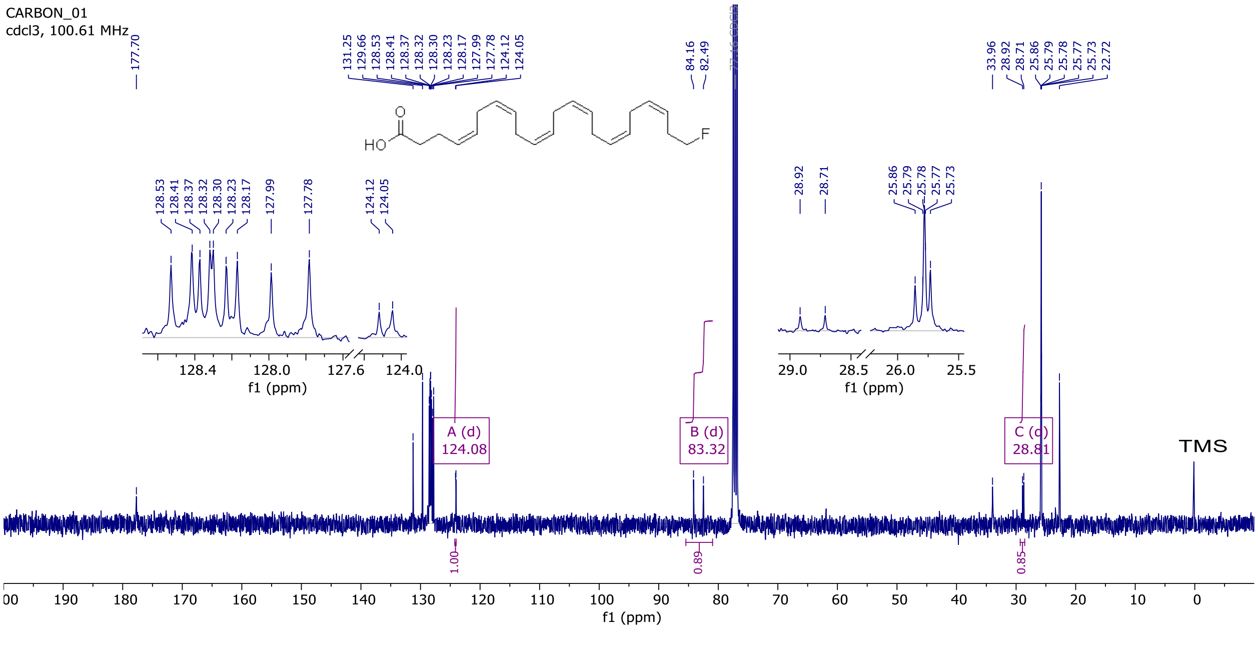

^{13}C{^{1}H} NMR ($CDCl_3$, 100 MHz) spectrum of 22-FDHA.

Ex Vivo LC-MS Analysis of 22-FDHA

Probe Injection and Tissue Extraction

A solution of 22-FDHA in acetonitrile was dried in a glass vial under a stream of filtered dry N_2 gas, and then under vacuum. The residue was formulated in injection buffer to a concentration of 10 ng/mL. A female mouse was anesthetized with isoflurane, and then 1 µg of 22-FDHA was injected into the tail vein. The mouse was kept under maintenance anesthesia for 30 min after which it was dissected and euthanized by cardiac puncture. Approximately 500 µL of blood was extracted from the heart and transferred to an EDTA-coated microcentrifuge tube. After blood collection, the mouse was immediately perfused with cold PBS then the liver and brain were extracted. Tissues, including isolated plasma, brain, and liver, were stored at –80 °C until processing. Following the same procedure, another mouse was injected with 500 ng 22-[^{18}F]FDHA, and the tissues were extracted at 30 min p.i.

Plasma and Brain 22-FDHA Extraction

22-FDHA was extracted from mouse plasma (100 µL) and brain tissue (~250 mg) and then quantified by liquid chromatography-tandem mass spectrometry (LC-MS/MS) using multiple reaction monitoring (MRM). Prior to extraction, mouse brains were cut in half through the midsagittal plane, and the left brain was used for analysis. Standard curve samples were prepared by spiking 3X stripped C57BL mouse plasma (BioIVT) with varied concentrations of synthetic 22-FDHA. To extract 22-FDHA and prevent lipid peroxidation, methanol (MeOH) containing 0.05% butylated hydroxytoluene and triphenylphosphine was added to plasma samples (200 µL) and brain tissues (500 µL). 50 µL d_5-DHA (50 ng/mL, Cayman) was added to each sample and used as the internal standard for quantification. To facilitate the extraction of 22-FDHA from plasma, samples were vortexed for 10 seconds and kept cool in ice for 10 min while brain tissues were homogenized. Brain samples were homogenized completely using a pre-cooled TissueLyser (Qiagen) at 30 Hz at 1.5-min intervals. Subsequently, plasma and brain samples were centrifuged at 10,000×*g* for 10 min at 4 °C. The supernatant was collected from each sample and diluted with water to obtain 10% MeOH solutions. Each sample was then subjected to solid phase extraction using Strata X 33µm Polymeric Reverse Phase cartridges (Phenomenex). Cartridges were pre-conditioned with MeOH (1 mL) followed by water (1 mL) before the diluted samples were eluted

through and then washed with water (1 mL). 22-FDHA and the internal standard were eluted using 700 µL MeOH, which was then evaporated to dryness under a steady stream of filtered dry N_2 gas. The dried residues were reconstituted with 50% aq. MeOH (50 µL) and centrifuged to remove any undissolved debris before transferring it to UV-resistant glass vials for LC-MS/MS analysis.

LC-MS/MS Analysis

22-FDHA and d_5-DHA were separated and quantified in an Agilent 1290 UPLC system coupled to a Sciex QTRAP API6500+ system using ESI in negative ion mode. For adequate separation, a Poroshell 120 EC-C_{18} column (2.7 µm, 4.6 × 100 mm, Agilent) was used with the mobile phases: A, 0.01% formic acid in H_2O; and B, 0.01% formic acid in MeOH. The gradient was as follows: 0–0.1 min, 20–50% B; 0.1–2 min, 50% B; 2–11 min, 50–80% B; 11–14.5 min, 80% B; 14.5–14.6 min, 80–98% B; 14.6–20 min, 98% B; 20–20.1 min, 98–20% B; 20.1–23 min, 20% B. An injection volume of 20 µL was used and the flow rate was held at 0.5 mL/min while the column temperature was set to 40 °C. Both 22-FDHA and d_5-DHA were detected using unique MRM signatures (Supplementary Table 1) and data analysis and quantification were performed using Skyline 23.1.[3]

Supplementary Table 1. LC-MS/MS Assay MRM Signatures

**Compound**	**Q1 (*m/z*)**	**Q3 (*m/z*)**	**RT (min)**	**DP (V)**	**CE (V)**	**CXP (V)**	**Internal Standard**
d_5-DHA	332.3	288.3	18.2	-60	-16	-14	N/A
22-FDHA	345.1	325.3	17.5	-80	-13	-14	d_5-DHA

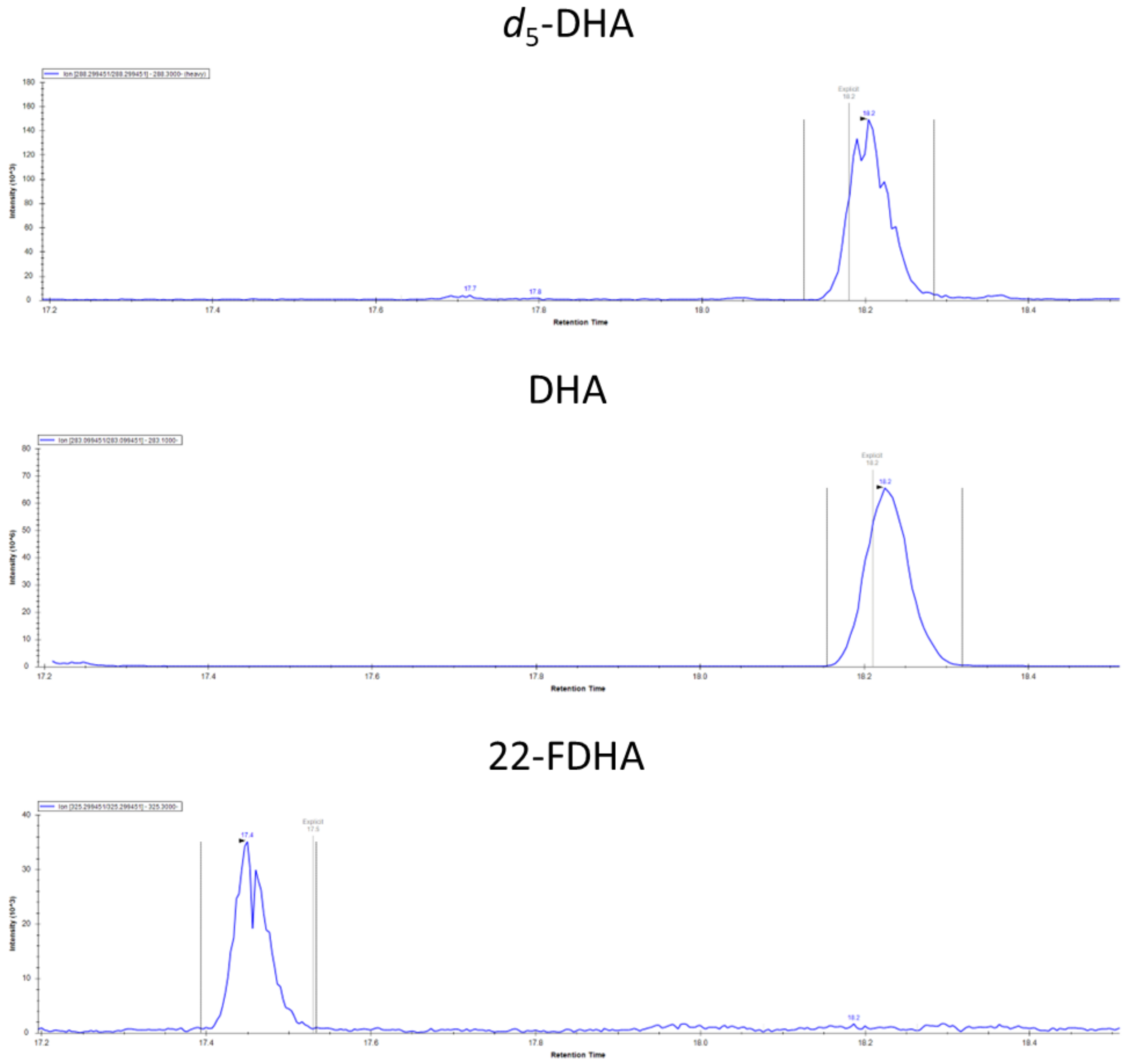

Supplementary Figure S1. Extracted ion chromatograms for *d*5-DHA (internal standard), DHA, and 22-FDHA of the brain extract of a mouse injected with 1 µg of 22-FDHA.

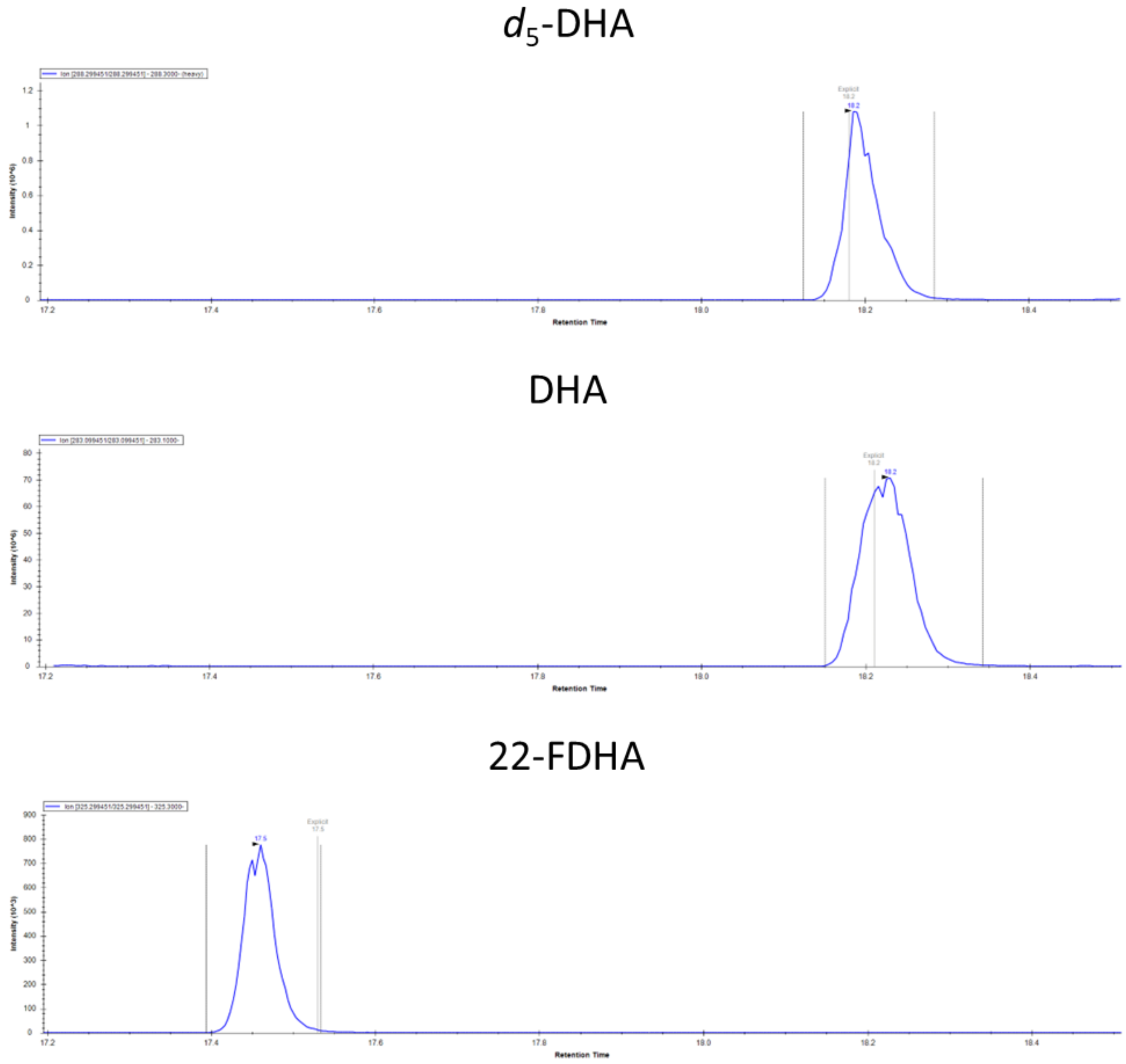

Supplementary Figure S2. Extracted ion chromatograms for d_5-DHA (internal standard), DHA, and 22-FDHA of the plasma extract of a mouse injected with 1 µg of 22-FDHA.

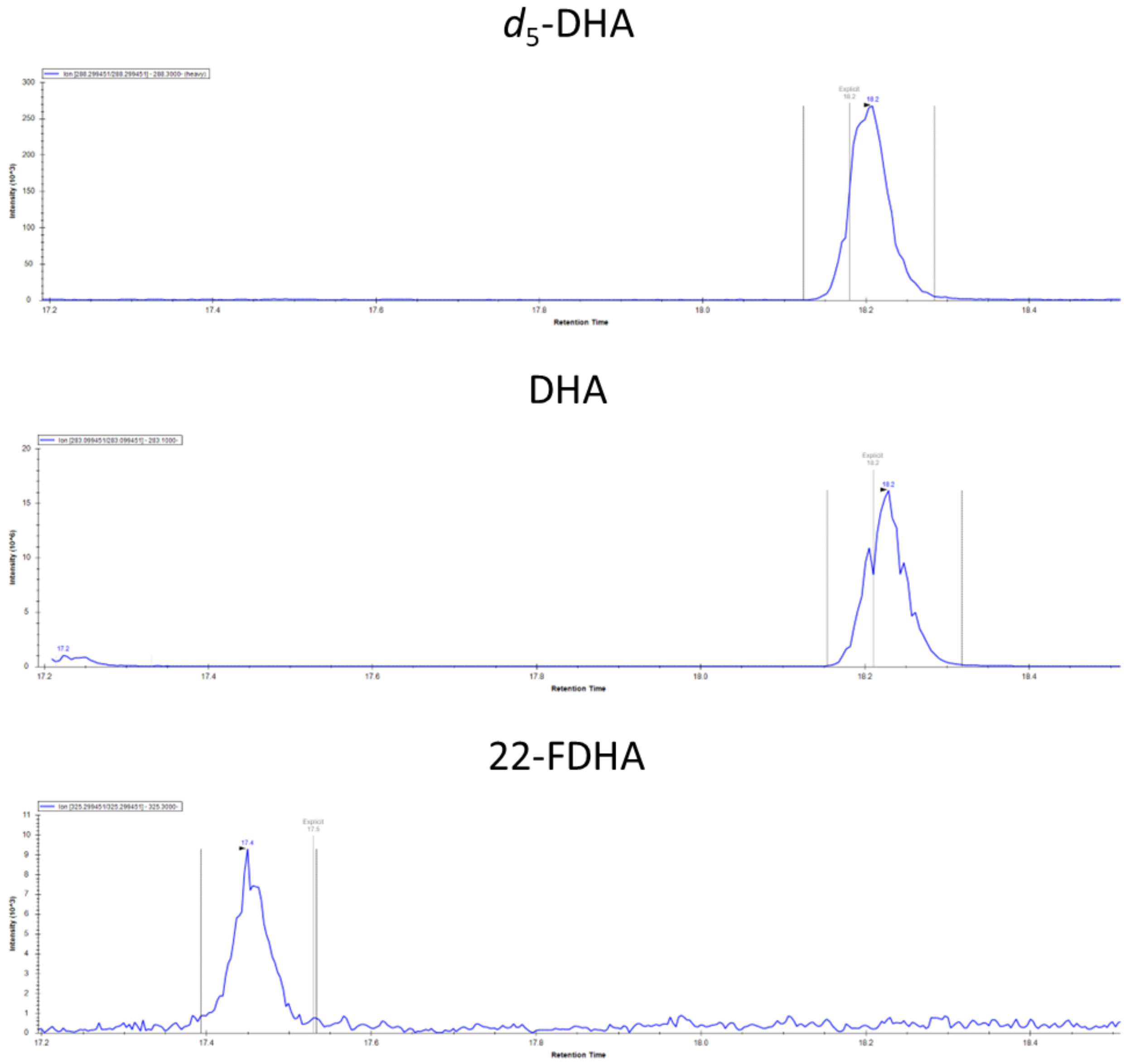

Supplementary Figure S3. Extracted ion chromatograms for *d*5-DHA (internal standard), DHA, and 22-FDHA of the liver extract of a mouse injected with 1 µg of 22-FDHA.

PET Imaging of 22-[^{18}F]FDHA in Normal Nude Mice

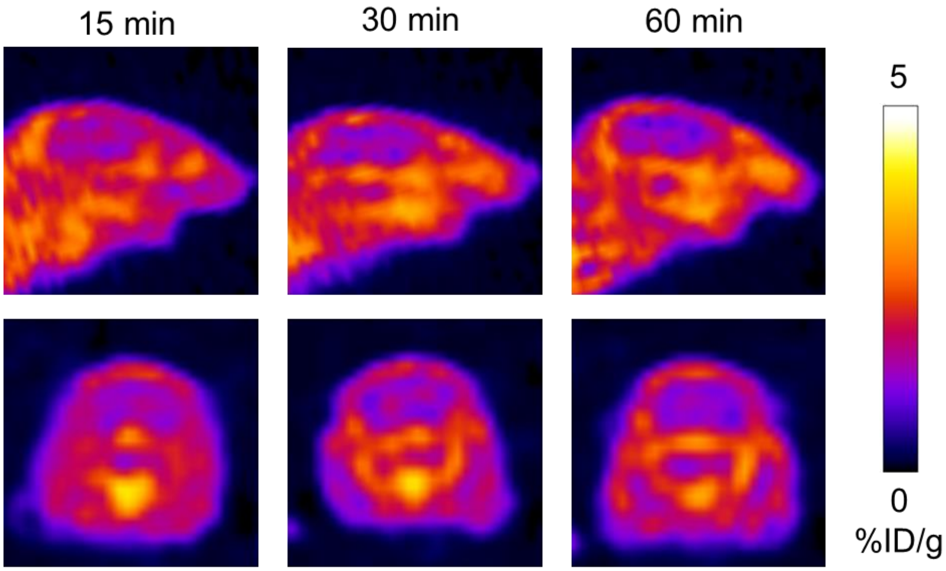

Supplementary Figure S4. PET images of a representative mouse after the injection of 22-[^{18}F]FDHA at different time points. Normal nude mice (n = 3) were injected with a bolus of 22-[^{18}F]FDHA (9–11 MBq) via the tail vein. PET images were acquired for 5 min at 15, 30, and 60 min p.i.

REFERENCES

(1) Hwang, S. H.; Wagner, K.; Xu, J.; Yang, J.; Li, X.; Cao, Z.; Morisseau, C.; Lee, K. S.; Hammock, B. D. Chemical Synthesis and Biological Evaluation of Omega-Hydroxy Polyunsaturated Fatty Acids. *Bioorg. Med. Chem. Lett.* **2017**, *27* (3), 620−625.

(2) Van Valkenburgh, J.; Duro, M. V. V.; Burnham, E.; Chen, Q.; Wang, S.; Tran, J.; Kerman, B. E.; Hwang, S. H.; Liu, X.; Sta Maria, N. S.; Zanderigo, F.; Croteau, E.; Rapoport, S. I.; Cunnane, S. C.; Jacobs, R. E.; Yassine, H. N.; Chen, K. Radiosynthesis of 20-[^{18}F]Fluoroarachidonic Acid for PET-MR Imaging: Biological Evaluation in ApoE4-TR Mice. *Prostaglandins Leukot. Essent. Fatty Acids* **2022**, *186*, 102510.

(3) Adams, K. J.; Pratt, B.; Bose, N.; Dubois, L. G.; St John-Williams, L.; Perrott, K. M.; Ky, K.; Kapahi, P.; Sharma, V.; MacCoss, M. J.; Moseley, M. A.; Colton, C. A.; MacLean, B. X.; Schilling, B.; Thompson, J. W.; Alzheimer's Disease Metabolomics, C. Skyline for Small Molecules: A Unifying Software Package for Quantitative Metabolomics. *J Proteome Res* **2020**, *19* (4), 1447−1458.
